# Ulvan Activates Chicken Heterophils and Monocytes Through Toll-Like Receptor 2 and Toll-Like Receptor 4

**DOI:** 10.3389/fimmu.2018.02725

**Published:** 2018-11-23

**Authors:** Nathalie Guriec, Frédérick Bussy, Christelle Gouin, Olivier Mathiaud, Benoit Quero, Matthieu Le Goff, Pi Nyvall Collén

**Affiliations:** ^1^Brest University and Brest Medical School, Brest, France; ^2^Amadeite SAS, Bréhan, France; ^3^R&D Breizh, Moustoir'ac, France

**Keywords:** ulvan, TLR, avian, heterophil, monocyte, inflammation

## Abstract

Responsiveness to invasive pathogens, clearance via the inflammatory response, and activation of appropriate acquired responses are all coordinated by innate host defenses. Toll-like receptor (TLR) ligands are potent immune-modulators with profound effects on the generation of adaptive immune responses. This property is being exploited in TLR-based vaccines and therapeutic agents in chickens. However, for administering the TLR agonist, all previous studies used *in ovo*, intra-muscular or intra-venous routes that cannot be performed in usual farming conditions, thus highlighting the need for TLR ligands that display systemic immune effects when given orally (*per os*). Here we have demonstrated that an ulvan extract of *Ulva armoricana* is able to activate avian heterophils and monocytes *in vitro*. Using specific inhibitors, we have evidenced that ulvan may be a new ligand for TLR2 and TLR4; and that they regulate heterophil activation in slightly different manner. Moreover, activation of heterophils as well as of monocytes leads to release pro-inflammatory cytokines, including interleukin1-β, interferon α and interferon γ, through pathways that we partly identified. Finally, when given *per os* to animals ulvan induces heterophils and monocytes to be activated *in vivo* thus leading to a transient release of pro-inflammatory cytokines with plasma concentrations returning toward baseline levels at day 3.

## Introduction

Over recent years, there has been an increase in the use of oral passive immunotherapy, in humans as well as in livestock, partly in both cases to reduce antibiotic therapy or prophylaxis. The crucial role and specificity of the innate immune response in driving and controlling adaptive immune responses to particular pathogens is now beginning to be understood and manipulated. It has been demonstrated that the Th1/Th2 paradigm applies in chicken (1). In the chicken, as in biomedical model species, Th1 cytokine response with interferon-gamma (IFNγ) release predominates in response to infection with intracellular pathogens like viruses, while Th2 cytokines such as interleukin 10 and 13 (IL10, IL13) are released in responses to infection with extracellular pathogens like bacteria. However, other cells than “classical” T cells have also recently been shown to produce IFNγ in response to vaccination against Newcastle disease in chickens (2). Cells of the avian innate immune system have been shown to recognize pathogens by pattern recognition receptors (PRR) that interact via pathogen associated molecular patterns (PAM). Toll-like receptor (TLR) ligands are potent immune-modulator with profound effects on the generation of adaptive immune responses. This property is being exploited in TLR-based vaccines and therapeutic agents in mammalian species and chickens (3). However, for administering the TLR agonist, all previous studies used *in ovo*, intra-muscular or intra-venous routes that cannot be performed in usual farming conditions, thus highlighting the need for TLR ligands that display systemic immune effects when given orally (*per os*).

Since some PRR ligands display polysaccharidic motifs (4) and since ulvans are polysaccharide chains with repeated motifs (5), we wondered whether ulvans may behave as chicken PRR agonist.

Therefore, the aim of the present study was to determine whether an ulvan extract of *Ulva armoricana* was able to activate avian heterophils and monocytes *in vitro*, to identify the PRR and the molecular pathways in the two cellular types and finally to determine whether a similar effect could be observed *in vivo* when given *per os*.

## Materials and methods

### Algal extract

Green tide algae *Ulva* sp. was collected on the beach at Plestin les Grèves (Bretagne, France; 48° 39′ 28^′′^ N, 3° 37′ 47^′′^ W) in June 2012. The algae were washed in fresh water, drained, and deep frozen. For extraction, the algae were thawed; wet ground, and liquid and solid phases were separated as part of an industrial process (Patent No. FR 61909). The liquid was fractionated by tangential filtration (Tami Industries). The protein content was evidenced with the BCA method (6), neutral sugars according to Dubois'method (7), uronic acids according to Blumenkrantz's (8), sugar-bound sulfates with Azure A quantification (9). Fatty acids were quantified according to an in house method adapted of the European directive CE152/2009, with a sensitivity threshold of 0.5 g/100 g. Lipids were also extracted with CHCl_3_/MeOH (2/1; v/v) and the amount of palmitic acid was quantified using GC-FID (gas chromatography with flame ionization detector) according to Le Croizier et al. (10). Fatty acids were identified by comparing their retention time with the ones of commercial standards. Comparing the fatty acids area with the one of the internal standard (C23:0) allowed their amount to be determined. The monosaccharide composition was determined by gas chromatography of trimethylsilyl derivatives, after acid methanolysis (MeOH/HCl 3N mixture) for 4 h (11). The molecular weight distribution of the sample (2 mg/ml) was analyzed by size exclusion chromatography in 0.1 M sodium nitrate with 0.2% sodium azide at a flow rate of 0.5 ml/min. The Shodex OHpak SB-806M HQ columns in series and a multi-angle light scattering refractometer (Wyatt, three angles) with a dn/dc of 0.142 ml/g were used for detection. In parallel the samples were dissolved on 99.97% atome D_2_O and subjected to RMN proton analysis. The RMN proton spectrum was registered at 298K on a Bruker Avance 500 spectrometer with a inversed cryogenic probe 5 mm _1_H/_13_C/_15_N TCI. The isotopic shifts were referenced with respect to an external standard (trimethylsilypropionic acid). No suppression of the HOD signal was performed. Lack of endotoxins was checked using the E-toxate^TM^ kit (Sigma), the detection threshold of which was 0.01 endotoxins Units/ml (1 pg/ml). The ulvan extract was dissolved under sterile conditions by gentle agitation for 6 h at 70°C in D-PBS buffered with 10 mM HEPES, filtered on a 0.2 μM filter and the supernatant autoclaved. Further dilutions were performed using RPMI-1640 medium (2 g/l glucose) buffered with 10 mM HEPES.

### Cell isolation

Sterile peripheral blood was obtained during routine follow-up of 28 days of age animals using heparin (20 U/ml) as anti-coagulant. The poultry veterinarians of the research team assessed the sanitary status before each experiment. Animals were raised without the use of antibiotics or any immune system stimulating chemicals from birth until blood sampling; the last vaccine was performed no later than day 12 of life for return to baseline immune parameters before the experiment. Blood from three chickens was pooled for each *in vitro* and *in vivo* experiment and cells purified as previously described (12). Briefly, blood was mixed with 1% methylcellulose (Sigma) in a 1.5:1 ratio and centrifuged at 25 g for 15 min. The supernatant was removed and suspended in Hanks' balanced salt solution without calcium or magnesium in a 1:1 ratio (Sigma). The suspension was then layered over a 1.077/1.119 Histopaque gradient (Sigma) and centrifuged at 250 g for 60 min. After centrifugation, the 1.077/1.119 interface containing mononuclear cells and the 1.119 band containing heterophils were collected separately and washed twice in RPMI-1,640 medium (2 g/l glucose, Sigma) supplemented with 10 mM HEPES (Sigma), further designed as complete medium (13–15). Cell viability was controlled by trypan blue staining and was typically >95%. The heterophils were immediately used for experiments. Mononuclear cells were suspended at 5 × 10^6^ cells/ml and allowed to adhere to plastic for 3 h at room temperature (100 μl per well in a 96 wells plate, 22°C). Non adherent cells were removed and adherent cells flushed to remove thrombocytes, the number of which was reduced to less than 5%, before incubation in complete medium followed by stimulation as described below.

### Cell stimulation

Production of an oxidative burst by heterophils was quantified by oxidation of the non-fluorescent DCFH-DA (Dichlorofluorescein-diacetate, Sigma) to fluorescent DCF (Dichlorofluorescein) as described previously (13) in five independent experiments carried out in triplicate. Briefly, 8 × 10^5^ heterophils were preincubated in a 100 μl volume at 41°C with 5% CO_2_ with DHFCA-DA (10 μg/ml final concentration) for 30 min prior to the addition of the ulvan extract. The oxidative burst induces the cleavage of this substrate and leads the liberated fluorescein emitting at 530 nm when excited at 485 nm. The Relative Fluorescent Units (RFU) were recorded as previously described (13).

Heterophils degranulation was measured in five independent experiments in triplicate by quantifying the amount of β-D-glucuronidase activity in the culture medium following stimulation of the heterophils as previously described (16). Heterophils (8 × 10^6^/ml) were incubated with each TLR agonist or ulvan extract for at least 1 h on a rocker platform at 41°C in a 5% CO_2_ incubator. The reaction was stopped by transferring the tubes containing the cells to an ice bath for 5–10 min. The cells were then centrifuged at 250 g, 10 min at 4°C. The supernatants were then removed and used for the assay. Thirty microliter aliquots of each supernatant were incubated in duplicate with 60 μl freshly prepared substrate (10 mM 4-methylumbelliferyl- β-D-glucuronide, 0.1% Triton X-100 in 0.1 M sodium acetate buffer) for 4 h at 41°C in a non-treated, black flat-bottom ELISA plate. The reaction was stopped by adding 200 μl of stop solution (0.05 M glycine and 5 mM EDTA; pH 10.4) to each well. Liberated 4-methylumbelliferone was measured fluorimetrically (excitation wavelength of 355 nm and an emission wavelength of 460 nm) with a fluorescence microplate reader. These values were converted to micromoles of 4-methylumbelliferone generated using a standard curve of known concentrations.

Monocytes were incubated with the ulvan extract in five independent experiments in duplicate. The cells were cultured in a RPMI-1640 glucose medium (2 g/l glucose) at 41°C with 5% of CO_2_, as previously described (13–15). The concentration of nitric oxide (NO) in conditioned media was determined in duplicate with Griess' reagent using a standard nitrite concentration curve.

All experiments on heterophils as well as on monocytes were carried out with glycogen as a negative control (10 μg/ml), LPS (Sigma) as TLR4 agonist (10 μg/ml), Pam3CSK4 as TLR2 agonist (10 μg/ml, Invivogen, further designed as PAM) as previously described (12, 13, 3) Glycogen was chosen as negative polysaccharide control to verify the specificity of the activation of the cells by ulvan. In order to identify the receptor(s) involved in ulvan's biological effects and to address the underlying mechanisms, specific inhibitors (Table 1) were added 30 min before the ulvan extract (25 μg/ml) in four independent experiments. Monocytes or heterophils were then incubated for 4 h as described above.

**Table 1:** Inhibitors and their relative targets.

**Name**	**Target**	**Final concentration**	**Manufacturer**
Chloroquinin	Cytoplasmic TLR (7/9/21)	100 μM	InvivoGen
2-aminopurine	TLR4/9/21 and PKR	5 mM	InvivoGen
OxPAPC	TLR2/4	50 μM	InvivoGen
Polymyxin B	TLR4	100 μM	InvivoGen
Anti-mTLR2-IgG	TLR2	0.66 nM	InvivoGen
YM201636	TLR9	5 μM	InvivoGen
Gefitinib	NOD (RIP2)	20 μM	InvivoGen
Piceatannol	Dectin	10 μM	InvivoGen
Glybenclamide	NLRP3	50 μM	InvivoGen
Parthenolide	NLRP1/3	40 μM	InvivoGen
Wortmannin	PI3K	40 nM	Sigma
Gö 6983	PKC	100 nM	Sigma
D609	PLC	100 μM	Sigma
SB203580	p38MAPK	40 μM	InvivoGen
SP600125	JNK	50 μM	InvivoGen
PD98059	ERK	200 μM	InvivoGen
Celastrol	NF-KB	10 μM	InvivoGen

### RNA extraction, RT-qPCR

Total RNA devoid of genomic DNA contamination was extracted with RNeasy Plus Mini Kit (Qiagen) and TRIzol® (Life Technologies) according to the manufacturers' instructions. Total RNA (200 ng) was used for first-strand cDNA synthesis with the High-Capacity cDNA Reverse Transcription kit (Applied Biosystems). RT-qPCR was performed using the Power SYBR Green PCR Master Mix (Applied Biosystems) for all transcripts. All determinations were performed in duplicate and normalized against actin as the internal control gene. The results are expressed as the relative gene expression with the DeltaDeltaCt method. Fold change = 2^−[(Ct target gene in sample−Ct actin in sample)−(Ct target gene in untreated cells^−^Ct actin in untreated cells)]^ (17). Primer sequences are listed in Supplemental Table 1. Variations were considered as significant when fold changes reached at least two.

### *in vivo* experiments

Three hundred male broiler chickens (28 days old at day 0) with a Ross 308 genetic background, obtained from a local commercial hatchery, were used in three independent experiments. This research was approved by the Brest University ethics committee in compliance with French laws and regulations. The experiments were conducted on adult animals to allow sufficient volume for blood sampling and in order to have fully functional heterophils (18). The last vaccination was performed no later than day 12 of life for return to baseline immune parameters before day 0 of the experiment. Blood samples (1 ml per animal) were taken every day from day 0 to day 3. All chickens received the same diet and prophylaxis programs within and between the experiments. All the animals were raised under standard farm conditions and had not been given any antibiotics or any immune system stimulating chemicals since birth. Each experiment consisted of four groups of 25 chickens. At day 0, around the first blood sampling, the animals were kept without water during 2 h to assure a homogeneous consumption of the extract. Each group then received a different dose of the ulvan extract in drinking water (0, 10, 25, 50 mg/l). At day 1 the solution was replaced by water. Chickens were placed in their respective pens (1 × 1.4 m allowing 0.56 cm^2^ of pen space per bird in accordance with French animal welfare laws) with straw, supplemental heat, water, and a balanced, un-medicated wheat and soybean based chicken diet *ad libitum*. Pelleted feed was given to the chickens twice daily at 09:00 a.m. and 05:00 p.m. The feed was formulated to contain 20% crude proteins and 3,200 kcal of metabolizable energy/kg of diet in agreement with the National Research Council and the genetic company (Aviagen) standards. Sanitary status, performance (mortality, daily average gain) and behavior were assessed daily, from 1 week before the experiment until slaughtering by the research team poultry veterinarians.

Individual quantification of plasma concentrations of glucuronidase activity and NO were performed as described above, while C-reactive protein (CRP), haptoglobin, interleukin1-β (IL1β), interferons α and γ (IFNα, IFNγ) concentrations were determined using ELISA kits as recommended by the manufacturer (Elabsciences). Heterophils and monocytes were purified as previously described to allow RT-qPCR experiments.

### Statistical analysis

Comparisons between groups were performed on at least three independent experiments for *in vivo* studies and five independent experiments for *in vitro* ones using ANOVA1 test and Bonferroni correction with the PRISM Software. Values are given as mean ± SEM. *P*-values of less than 0.05 were considered significant.

## Results

### Characteristics of the ulvan extract

**Figure 1:**
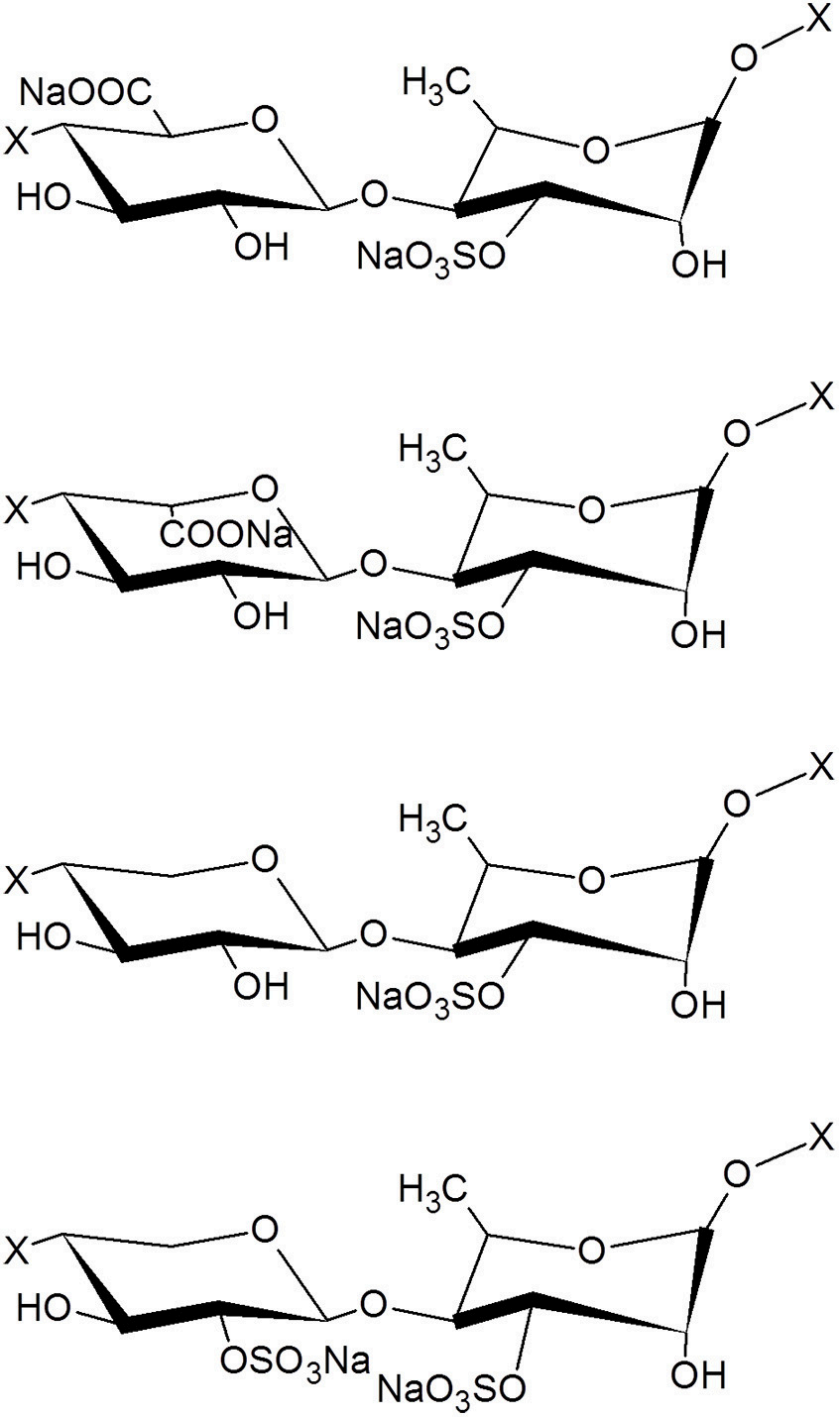
Structure of the main disaccharide motifs present in ulvan. From the top to the bottom: β(1,4)-D-GlcA-α (1,4)-L-Rha 3 sulfate, β(1,4)-L-idoA-α (1,4)-L-Rha 3 sulfate, β(1,4)-D-xyl-α (1,4)-L-Rha 3 sulfate, β(1,4)-D-xyl 2-sulfate-α(1,4)-L-Rha 3 sulfate, where X represents the continuation of the polysaccharide chain. GlcA, glucuronic acid; Rha, rhamnose; IdoA, iduronic acid; Xyl, xylose.

The protein content in the dry matter was 8.9 ± 0.3%. No fatty acids and no endotoxins could be detected. The average contents were 40.2 ± 0.7% for neutral sugars, 32.2 ± 0.8% for uronic acids, 8.3 ± 0.3% for sugar-bound sulfates. The monosaccharide composition evidenced the characteristic ulvan composition with, rhamnose, xylose, iduronic acid, and glucuronic acid (Figure 1). The weight-average (Mw) and the number-average (Mn) molecular weight were estimated as 5.8 ± 0.6 and 3.4 ± 0.3 kDa, respectively, and the polydispersity index was calculated to be 1.57 ± 0.03. Moreover, the proton NMR analysis showed a profile very closed to the one obtained of oligosaccharides from an ulvan (Supplemental Figure 1).

### Ulvan activates heterophils

**Figure 2:**
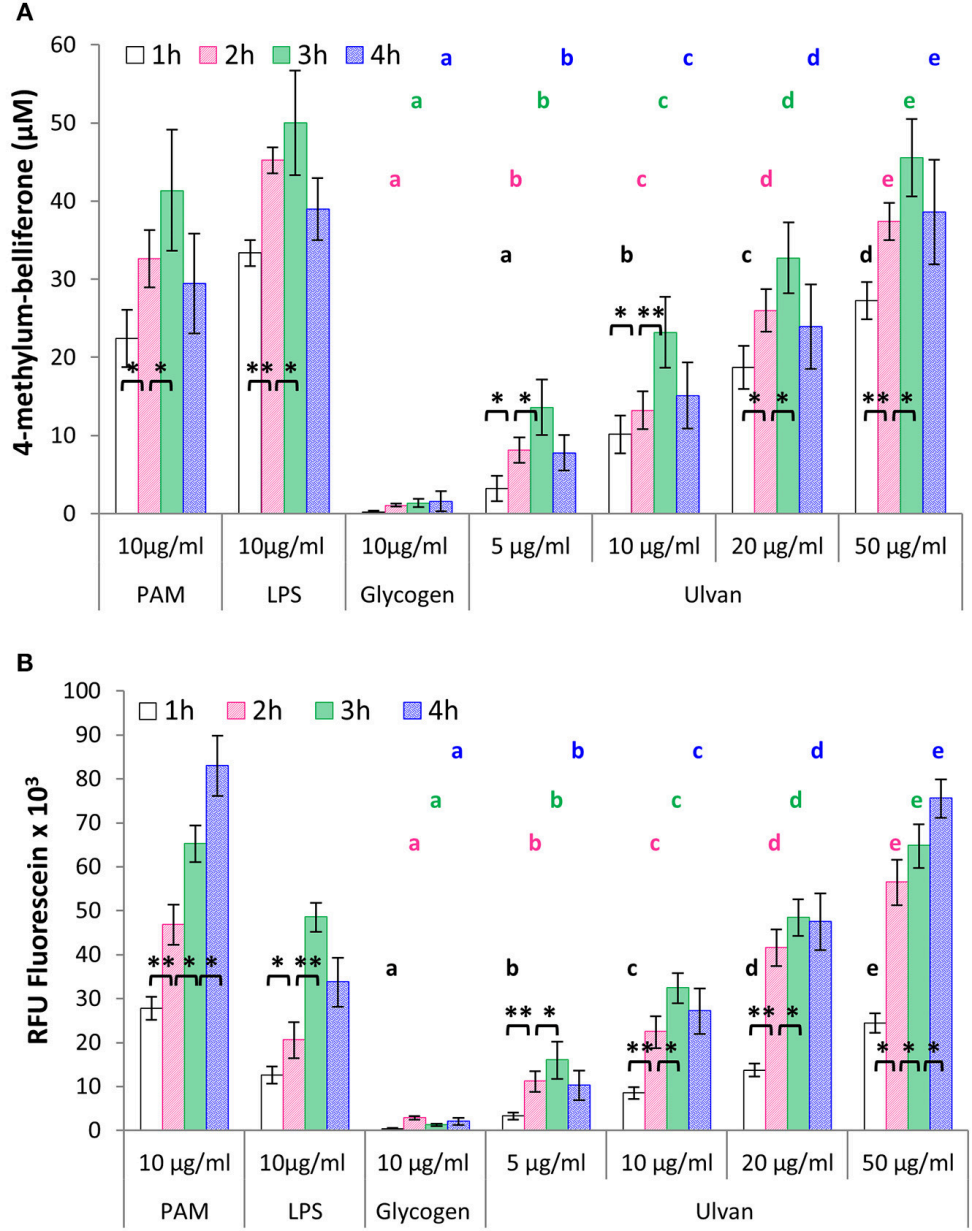
Ulvan activates heterophils *in vitro* in a time- and dose-dependent manner. Heterophils (8 × 10^6^/ml) were incubated *in vitro* with different concentrations of ulvan, PAM as TLR2 agonist, LPS as TLR4 agonist, and glycogen as a control of polysaccharide specificity. Activation was evidenced by the quantification of degranulation as evidenced by β-D-glucuronidase ability to generate 4-methylum-belliferone **(A)**; and the measurement of the oxidative burst as evidenced by fluorescein fluorescence **(B)**. Data represents the mean ± SEM of five independent experiments in triplicate. *p < 0.05, **p < 0.01 for statistically different values for the same dose at different times. Different letters with the same color indicate statistically different values for the different doses at the same time (*p* < 0.05).

Heterophils are considered as the poultry equivalents of mammalian neutrophils, and as such an integral part of the avian innate defenses against pathogens. Incubation with the ulvan extract leads to glucuronidase release by heterophils in a dose-dependent manner, with a peak at 3 h of incubation, as also observed for the positive controls LPS and PAM, but not the negative one, glycogen (Figure 2A). Four-methylum-belliferone concentrations at *t* = 3 h were, 1.41 ± 0.49 μM with medium alone, 1.34 ± 0.52 μM with 10 μg/ml glycogen, 23.19 ± 4.54 μM with 10 μg/ml ulvan, 32.73 ± 4.56 μM with 20 μg/ml, 45.54 ± 4.93 μM with 50 μg/ml, 50.00 ± 6.70 μM with 10 μg/ml LPS, 41.36 ± 7.74 μM with 10 μg/ml PAM. Moreover incubation with the ulvan also induced an oxidative burst by the heterophils, as evidenced by the fluorescein release (Figure 2B). The highest ulvan concentration (50 μg/ml) resulted in a maximum burst after 4 h of incubation (75.53 × 10^3^ ± 4.41 × 10^3^RFU) similar to the one observed for the TLR2 agonist PAM, but superior to the one of LPS, the TLR4 agonist (33.73 × 10^3^ ± 5.60 × 10^3^RFU). As for LPS, the lower doses of ulvan resulted in a maximal stimulation at 3 h that would tend to decrease in a non-statistically significant manner at 4 h (Figure 2B).

### Heterophil activation is TRL2/4 dependent

Using selective inhibitors (Table 1) we analyzed the capacity of ulvan (25 μg/ml) to act as a PRR agonist. Blocking TLR2 on neutrophils resulted in a statistically significant decrease in degranulation (Table 2) and oxidative burst (Table 2). Similarly, TLR4 blockade resulted in a statistically significant decrease in degranulation and in the oxidative burst. Interestingly, the action of ulvan on these receptors have a synergistic effect since the decrease obtained was 89.92 ± 1.34% for the degranulation and 90.55 ± 4.52% for the burst, when both TLR2 and TLR4 were blocked *p* < 0.05 (Table 2).

**Table 2:** Heterophils are activated through TLR2 and TLR4 through PKC and PLC dependent mechanisms.

**Inhibitor target**	**Remaining percentage of 4-methylum-belliferone release**	**Statistical significance**	**Remaining percentage of RFU Fluorescein**	**Statistical significance**
No inhibition	100.00		100.00
TLR2	48.54 ± 3.98	*p* < 0.01	44.75 ± 2.49	*p* < 0.01
TLR4	52.08 ± 5.15	*p* < 0.01	57.43 ± 3.38	*p* < 0.01
TLR2+4	10.08 ± 1.34	*p* < 0.005	9.45 ± 4.52	*p* < 0.005
TLR4+21	50.08 ± 6.11	*p* < 0.01	48.58 ± 3.44	*p* < 0.01
TLR21	98.47 ± 4.35	None	94.04 ± 2.16	None
Cytoplasmic TLR	96.45 ± 3.17	None	96.58 ± 2.02	None
NOD	95.61 ± 3.85	None	98.52 ± 2.35	None
Dectin	98.72 ± 3.26	None	96.43 ± 3.54	None
NLRP3	94.24 ± 3.49	None	95.31 ± 2.81	None
NLRP1+3	98.80 ± 3.75	None	98.84 ± 2.64	None
PKC	45.783 ± 3.26	*p* < 0.01	59.73 ± 4.91	*p* < 0.01
TLR2 + PKC	18.19 ± 4.11	*p* < 0.005	28.59 ± 3.16	*p* < 0.005
TLR4 + PKC	24.01 ± 5.22	*p* < 0.005	30.97 ± 2.89	*p* < 0.005
PLC	58.15 ± 3.51	*p* < 0.01	56.06 ± 4.99	*p* < 0.01
TLR2 + PLC	52.99 ± 4.88	*p* < 0.01	25.63 ± 3.95	*p* < 0.005
TLR4 + PLC	23.15 ± 5.44	*p* < 0.005	25.88 ± 3.34	*p* < 0.005
PI3K	64.85 ± 2.79	*p* < 0.05	79.07 ± 5.48	None
p38MAPK	90.82 ± 2.92	None	86.96 ± 4.74	None
JNK	90.62 ± 4.67	None	84.70 ± 5.84	None
ERK	88.41 ± 2.49	None	85.49 ± 3.59	None
NF-KB	86.68 ± 2.98	None	77.69 ± 9.04	None

*Heterophils (8 × 10^6^/ml) were first incubated for 30 min with inhibitors specific for different PRRs' or intracellular proteins before addition of the ulvan extract at 25 μg/ml for 4 h. Degranulation was evidenced by the remaining percentage of 4-methylum-belliferone release, the oxidative burst by the remaining percentage of Fluorescein Relative Fluorescent Units (RFU). Data represents the mean ± SEM of four independent experiments in triplicate*.

We then investigated the intracellular proteins required for the degranulation and the oxidative burst. As shown in Table 2, p38MAPK, JNK, ERK, NF-κB did not regulate these events since their inhibition did not result in a statistically significant decrease. The more potent inhibitors for degranulation appeared to be those against PI3K, PKC, and PLC (Table 2). Moreover, PKC and PLC, but not PI3K, appeared to be major regulators of the oxidative burst (Table 2). In addition, PKC and PLC acted on both TLR4 and TLR2 pathways, in an equal manner for the oxidative burst but not for degranulation with the TLR4-mediated oxidative burst which appears as independent from PLC (Table 2).

### Ulvan triggers cytokine transcription in heterophils

The transcription pattern of IL1β, IFNα, IFNγ, IL8, and IL18 varied in a dose- and time-dependent manner in response to the ulvan extract (Table 3). Variations appeared as soon as 2 h of incubation. The fold changes were specially increased for IL1β, IFNα, and IFNγ while transcription of IL8 and IL18 genes was induced in a lesser extent. No significant variation was observed for IFNβ, IL10, IL13, and IL17 and no negative fold changes were observed (Supplemental Table 2).

**Table 3:** Ulvan triggers cytokine transcription in heterophils.

**Gene**	**Time**	**No addition**	**Glycogen 10 μg/ml**	**Ulvan 10 μg/ml**	**Ulvan 25 μg/ml**	**Ulvan 50 μg/ml**	**Statistical difference between *t* = 2 h and *t* = 4 h**
**FOLD CHANGES, HETEROPHILS**							
IL1β	2 h	1.01 ± 0.11	1.00 ± 0.09^a^	5.10 ± 0.46^b^	17.10 ± 1.76^c^	41.02 ± 3.60^d^	*p* < 0.05 for ulvan 25 μg/ml and 50 μg/ml
	4 h	0.99 ± 0.08	0.94 ± 0.09^a^	6.35 ± 0.70^b^	31.27 ± 3.83^c^	92.71 ± 9.53^d^
IFNα	2 h	1.06 ± 0.12	1.00 ± 0.14^a^	2.24 ± 0.03^b^	4.32 ± 0.31^c^	17.61 ± 1.29^d^	*p* < 0.05 for ulvan 25 μg/ml and 50 μg/ml
	4 h	0.94 ± 0.09	0.98 ± 0.10^a^	2.70 ± 0.26^b^	16.43 ± 1.52^c^	63.29 ± 5.67^d^
IFNγ	2 h	1.09 ± 0.09	1.00 ± 0.10^a^	6.51 ± 0.54^b^	9.57 ± 1.02^c^	37.60 ± 2.81^d^	*p* < 0.01 for ulvan 25 μg/ml and 50 μg/ml
	4 h	0.96 ± 0.10	0.97 ± 0.09^a^	6.02 ± 0.69^b*^	67.28 ± 6.50^#^	294.94 ± 31.22^&^
IL8	2 h	0.97 ± 0.05	1.02 ± 0.02^a^	2.43 ± 0.18^b^	4.28 ± 0.26^c^	5.54 ± 0.44^d^	None
	4 h	1.05 ± 0.11	1.07 ± 0.10^a^	2.06 ± 0.22^b^	3.81 ± 0.36^c^	6.38 ± 0.63^d^
IL18	2 h	1.02 ± 0.11	0.98 ± 0.10	1.20 ± 0.15	1.26 ± 0.12^a^	2.27 ± 0.19^b^	*p* < 0.05 for ulvan 25 μg/ml and 50 μg/ml
	4 h	0.98 ± 0.09	0.97 ± 0.10^a^	1.35 ± 0.12^b^	2.57 ± 0.26^c^	5.41 ± 0.55^d^
TLR2	2 h	1.05 ± 0.09	1.01 ± 0.10	1.13 ± 0.94^a^	2.01 ± 0.13^b^	4.61 ± 0.32^c^	*p* < 0.05 for ulvan 25 μg/ml and 50 μg/ml
	4 h	0.99 ± 0.09	0.97 ± 0.10^a^	1.68 ± 0.12^b^	4.91 ± 0.54^c^	13.24 ± 1.30^d^
TLR4	2 h	0.99 ± 0.08	1.00 ± 0.10	1.25 ± 0.09	0.96 ± 0.09^a^	2.62 ± 0.20^b^	*p* < 0.05 for ulvan 25 μg/ml and 50 μg/ml
	4 h	1.00 ± 0.09	0.97 ± 0.08	1.15 ± 0.09^a^	2.77 ± 0.21^b^	7.58 ± 0.68^c^

*Variations are expressed as mRNA fold changes using β-actin as an internal standard. Transcription is time- and dose-dependent. Data represents the mean ± SEM of five independent experiments in triplicate. Different letters indicate statistically significant differences (a, b, c, and d) with p < 0.05 for the same incubation time, different symbols mean statistically significant differences with p < 0.01 for the same incubation time*.

Heterophil activation also resulted in raised transcription of TLR2 and TLR4 receptors, with TLR2 being the most affected (Table 3). The use of specific inhibitors confirmed that the variations in cytokine transcription are the results of TLR2 and/or TLR4 activation and that TLR2 and TLR4 act synergistically to regulate these genes (Table 4). Moreover, TLR2 and TLR4 genes appear to be the downstream targets of their own receptors. In addition each one of them regulates its own transcription but also the one of the other TLR (Table 4). Four hours after the application of the ulvan extract, TLR2 and TLR4 seem to regulate their transcription through mechanisms involving NF-κB (*p* < 0.05) as transcriptional activator and PI3K as a repression inducer (*p* < 0.01, Table 4). PKC and PLC inhibitors had no effect on the fold changes of any of the genes whose expression was increased with the extract. P38MAPK and JNK inhibitor also had no impact on IFNα, IFNγ, TLR2, and TLR4 genes transcription. Meanwhile, p38MAPK and JNK inhibition resulted in the reduction of IL1β, IL8, and IL18 transcription. In addition, NF-κB and ERK activated IL1β, IFNα, IFNγ, IL8, and IL18 transcription to a comparable extent (Table 4). We next wondered whether these proteins were activated following the addition of the extract and the subsequent activation of TLR2 and TLR4. We thus performed simultaneous inhibition experiments using TLR inhibitors in combination with cytoplasmic targets ones and focused on genes which expression was modified after the addition of the ulvan extract (Table 4). Both TLR2 and TLR4 pathways appear to involve NF-κB and ERK as regulators of IL1β, IFN α, IFNγ, IL8, and IL18 genes transcription. PLC, PKC, PI3K did not significantly affect the transcription rate of these genes (Table 5). In addition, the transcription of IFNα and IFNγ genes is also not significantly regulated by JNK, p38MAPK (Table 5). TLR2 and TLR4 receptors display a regulation pattern involving for each one of them NF-κB as a transcriptional activator and PI3K as a transcriptional repressor (Table 5).

**Table 4:** Transcription in heterophils is TLR2 and TLR4 dependent and involves intracellular mediators.

**Inhibitor target**	**IL1β**	**IFNα**	**IFNγ**	**IL8**	**IL18**	**TLR2**	**TLR4**
**FOLD CHANGES, HETEROPHILS**							
No inhibitor	46.53 ± 1.64	22.12 ± 1.57	88.75 ± 3.49	4.08 ± 0.11	3.74 ± 0.16	6.56 ± 0.32	5.13 ± 0.33
TLR2	18.44 ± 2.57^b^	11.41 ± 0.99^b^	48.46 ± 1.19^c^	1.70 ± 0.15^a^	1.67 ± 0.19^a^	2.37 ± 0.15^a^	2.54 ± 0.14^a^
TLR4	13.81 ± 1.45^b^	10.27 ± 0.26^b^	48.29 ± 0.75^c^	1.93 ± 0.18^a^	1.64 ± 0.08^a^	2.22 ± 0.21^a^	3.26 ± 0.28^a^
TLR2+4	1.46 ± 0.13^c^	1.15 ± 0.12^c^	1.14 ± 0.15^d^	0.96 ± 1.10^b^	0.92 ± 0.10^b^	2.14 ± 0.27^a^	2.61 ± 0.19^a^
TLR4+21	11.59 ± 1.08^b^	10.67 ± 0.24^b^	49.97 ± 0.76^c^	1.95 ± 0.25^a^	1.73 ± 0.17^a^	2.50 ± 0.03^a^	3.08 ± 0.04^a^
TLR9	47.60 ± 1.46	21.61 ± 0.93	83.65 ± 6.98	4.015 ± 0.23	3.48 ± 0.26	6.51 ± 0.23	5.07 ± 0.38
Cytoplas mic TLR	47.43 ± 2.11	20.56 ± 2.25	91.73 ± 5.58	3.87 ± 0.2	3.51 ± 0.20	6.12 ± 0.28	5.27 ± 0.15
NOD	46.75 ± 2.00	19.86 ± 1.13	88.51 ± 4.14	4.096 ± 0.28	3.59 ± 0.12	6.24 ± 0.42	5.16 ± 0.06
Dectin	47.21 ± 2.58	21.79 ± 1.61	88.03 ± 3.48	4.029 ± 0.05	3.88 ± 0.41	6.33 ± 0.33	5.28 ± 0.40
NLRP1+3	47.18 ± 2.54	22.97 ± 1.82	86.16 ± 4.20	4.10 ± 0.21	3.83 ± 0.29	6.59 ± 0.21	5.18 ± 0.28
NLRP3	47.42 ± 2.22	21.70 ± 2.42	85.59 ± 3.85	3.86 ± 0.26	3.79 ± 0.29	6.32 ± 0.22	4.43 ± 0.75
NF-KB	6.2 ± 0.64^b^	15.52 ± 1.19^a^	64.23 ± 7.91^a^	1.84 ± 0.22^a^	1.81 ± 0.13^a^	2.43 ± 0.19^a^	1.72 ± 0.13^a^
ERK	7.51 ± 0.55^b^	15.59 ± 1.57^a^	66.51 ± 6.45^a^	1.95 ± 0.10^a^	2.06 ± 0.21^a^	6.10 ± 0.59	4.36 ± 0.40
JNK	2.80 ± 0.34^b^	18.46 ± 1.79	89.81 ± 7.49	1.29 ± 0.14^a^	1.24 ± 0.11^a^	6.63 ± 0.66	4.17 ± 0.38
p38 MAPK	2.92 ± 0.04^b^	22.18 ± 1.96	85.79 ± 9.62	1.29 ± 0.14^a^	1.42 ± 0.17^a^	4.2 ± 0.31	3.77 ± 0.32
PLC	48.44 ± 4.83	22.1 ± 1.65	83.52 ± 8.20	4.32 ± 0.41	4.35 ± 0.37	4.01 ± 0.42	3.36 ± 0.29
PKC	48.83 ± 3.39	23.54 ± 1.78	79.41 ± 7.06	1.07 ± 0.08	4.29 ± 0.46	3.86 ± 0.37	3.39 ± 0.34
PI3K	32.23 ± 2.34	23.69 ± 1.96	89.94 ± 8.40	4.06 ± 0.35	3.60 ± 0.35	16.87 ± 1.48^b^	13.56 ± 1.66^b^

Variations are expressed as mRNA fold changes using β-actin as an internal standard. Heterophils (8 × 10^6^/ml) were first incubated for 30 min with the inhibitors before addition of the ulvan extract at 25μg/ml for 4 h. Data represents the mean ± SEM of four independent experiments in triplicate. Letters indicate statistically significant differences with the control without inhibitor,

a p < 0.05,

b p < 0.01,

c p < 0.005,

d *p < 0.001*.

**Table 5:** TLR2 and TLR4 pathways regulate transcription in heterophils through common intracytoplasmic mediators.

**Inhibitor target**	**IL1β**	**IFNα**	**IFNγ**	**IL8**	**IL18**	**TLR2**	**TLR4**
**FOLD CHANGES, HETEROPHILS**							
No inhibitor	43.53 ± 3.51	21.33 ± 1.405	88.03 ± 1.89	4.31 ± 2.93	3.76 ± 0.36	6.40 ± 0.59	5.14 ± 0.56
TLR2	20.04 ± 2.41	11.76 ± 0.70	45.93 ± 4.42	1.84 ± 0.371	2.15 ± 0.22	2.71 ± 0.26	2.56 ± 0.11
TLR2 + NFKB	7.21 ± 0.54^a^	6.18 ± 0.56^a^	25.81 ± 1.71^a^	1.08 ± 0.11^a^	1.03 ± 0.11^a^	1.23 ± 0.18^a^	1.45 ± 0.15^a^
TLR2 + ERK	7.50 ± 0.22^a^	7.81 ± 0.65^a^	26.49 ± 1.97^a^	1.00 ± 0.03^a^	0.97 ± 0.10^a^	1.97 ± 0.18	3.44 ± 0.27
TLR2 + JNK	3.45 ± 0.27^b^	11.42 ± 0.98	41.81 ± 2.63	0.99 ± 0.07^a^	0.45 ± 0.03^b^	2.39 ± 0.24	3.65 ± 0.21
TLR2 + p38	3.11 ± 0.14^b^	10.69 ± 0.82	45.14 ± 5.36	0.91 ± 0.05^a^	0.53 ± 0.05^b^	2.60 ± 0.30	3.83 ± 0.28
TLR2 + PLC	19.41 ± 1.60	10.43 ± 1.29	42.46 ± 3.42	1.50 ± 0.08	1.87 ± 0.15	2.60 ± 0.23	3.23 ± 0.31
TLR2 + PKC	19.72 ± 0.71	12.19 ± 1.16	43.52 ± 3.91	1.45 ± 0.12	1.78 ± 0.68	2.59 ± 0.27	3.08 ± 0.30
TLR2 + PI3K	16.44 ± 0.44	11.33 ± 0.77	45.16 ± 4.13	1.50 ± 0.16	1.71 ± 0.13	4.04 ± 0.41^a^	5.57 ± 0.49^a^
TLR4	14.21 ± 0.80	10.46 ± 1.12	48.62 ± 4.17	1.86 ± 0.20	1.74 ± 0.18	2.41 ± 0.19	2.70 ± 0.21
TLR4 + NFKB	5.78 ± 0.47^a^	5.34 ± 0.48^a^	27.13 ± 2.32^a^	0.51 ± 0.03^b^	0.71 ± 0.06^a^	1.32 ± 0.14^a^	1.01 ± 0.10^a^
TLR4 + ERK	7.62 ± 0.44^a^	6.55 ± 0.72^a^	22.36 ± 1.28^a^	0.53 ± 0.04^b^	0.73 ± 0.06^a^	1.85 ± 0.18	2.49 ± 0.22
TLR4 + JNK	3.25 ± 0.31^b^	9.86 ± 0.86	41.62 ± 3.89	0.53 ± 0.04^b^	0.81 ± 0.08^a^	2.29 ± 1.17	2.07 ± 0.21
TLR4 + p38	2.01 ± 0.13^b^	9.68 ± 0.86	43.38 ± 4.11	0.61 ± 0.06^b^	0.49 ± 0.04^b^	2.14 ± 0.22	2.43 ± 0.21
TLR4 + PLC	13.44 ± 0.88	10.24 ± 1.01	44.05 ± 4.29	1.61 ± 0.17	1.75 ± 0.18	1.97 ± 0.20	2.22 ± 0.18
TLR4 + PKC	13.21 ± 0.65	10.43 ± 0.96	46.20 ± 3.96	1.63 ± 0.21	1.55 ± 0.17	2.27 ± 0.20	2.17 ± 0.18
TLR4 + PI3K	13.03 ± 0.76	11.76 ± 1.31	46.71 ± 4.55	1.48 ± 0.12	1.67 ± 0.14	4.43 ± 0.43^a^	6.98 ± 0.61^a^

Variations are expressed as mRNA fold changes using β-actin as an internal standard. Heterophils (8 × 10^6^/ml) were first incubated for 30 min with the inhibitors before addition of the ulvan extract at 25 μg/ml for 4 h. Data represents the mean ± SEM of four independent experiments in triplicate. Letters indicate statistically significant differences with the experiment with the TLR inhibitor,

a p < 0.05,

b *p < 0.01*.

### Ulvan causes no release by monocytes in a TLR2/4 -dependent manner *in vitro*

**Figure 3:**
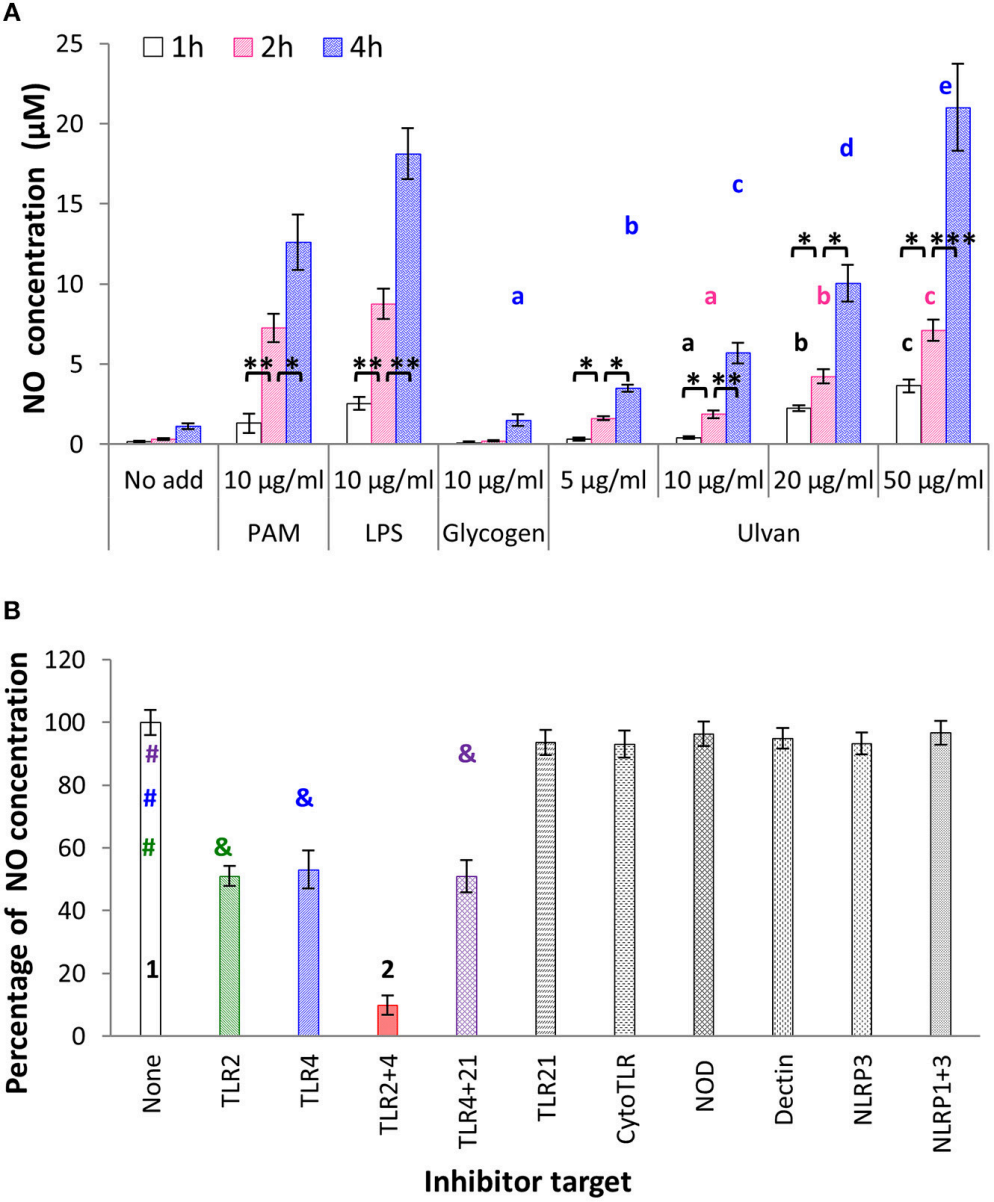
**(A)** Ulvan causes NO release by monocytes. Ulvan triggers NO release in a dose- and time dependent manner, measured using Griess' reagent. Data represents the mean ± SEM of four independent experiments in duplicate. Letters indicate statistically different values for the different doses at the same time with *p* < 0.05. *p < 0.05, **p < 0.01, ***p < 0.005 when values are statistically different for the same dose at different times. **(B)** Ulvan acts through TLR2- and TLR4-dependent mechanisms. Monocytes were incubated *in vitro* with different concentrations of ulvan, PAM as TLR2 agonist, LPS as TLR4 agonist, and glycogen as a control of polysaccharide specificity. The monocytes were first incubated for 30 min with the inhibitors before addition of the ulvan extract at 25 μg/ml for 4 h. Data represents the mean ± SEM of four independent experiments in duplicate. Different symbols with the same color indicate statistically different values with *p* < 0.01, different numbers mean statistically different values with *p* < 0.005.

Another leader cell in innate immunity is the monocyte, due to its ability to synthesize NO and cytokines, and to link innate and adaptive immunity (19). When incubated with ulvan, monocytes secreted statistically significant amounts of NO (Figure 3A). NO release was time- and dose-dependent, with a maximal effect observed after 4 h of incubation, and for 50 μg/ml ulvan. Under these conditions NO concentrations rose to 1.12 ± 0.18 μM with medium alone, 1.49 ± 0.36 μM with glycogen, 3.49 ± 0.21 μM with 5 μg/ml ulvan, 5.69 ± 0.63 μM with 10 μg/ml ulvan, 10.05 ± 1.16 μM with 20 μg/ml ulvan, 21.03 ± 2.71 μM with 50 μg/ml ulvan and for the two positive controls, 12.62 ± 1.73 μM with PAM, 18.12 ± 1.58 μM with LPS. As observed for heterophils, TLR2 and TLR4 are the main membrane targets of ulvan as their blockade resulted in reduction by half of the NO concentration, respectively, and a 90.09 ± 2.35% decrease when simultaneously blocked (*p* < 0.05, Figure 3B).

### Cytokines and iNOS genes are transcribed in monocyte after TLR2/4 activation

The transcription of IFNβ, IL10, IL17, and IL18 genes was not affected by ulvan (Supplemental Table 3). However, variation in gene transcription was observed for IL1β, IFNα, IFNγ, IL8, IL13, TLR2, TLR4, and iNOS, in a dose-dependent manner (Table 6). The induction was also time-dependent, except for IL8 whose fold changes were very similar between the two times, with for instance with 50 μgm/l ulvan 4.06 ± 0.4 at 2 h, 3.75 ± 0.43 at 4 h (Table 6). As noted for heterophils, the induction was especially strong for IL1β, IFNα, and IFNγ. However, in contrast to what was observed in heterophils the transcription of IL1β and IFNα was not significantly modified at 2 h with a fold change around 1. IL1β transcription was only tuned on after 4 h with 25 and 50 μg/ml (Table 6). TLR2 and TLR4 receptors are responsible for the variations in the fold changes since their blockade severely hampers the transcription of IL1β, IFNα, IFNγ, IL8, IL13, iNOS, TLR2, and TLR4 (Table 7). TLR2 and TLR4 act, at least in part through NF-κB activation, since NF-κB inhibition results in a reduction of the positive fold changes for all these genes (Table 7). NF-κB appears to be the unique transcriptional activator for IFNα and IL13 among the ones we investigated, while IFNγ transcription is also increased by ERK (Table 7). As observed in heterophils, ERK and JNK act as transcriptional activators for IL1β and IL8 genes and p38MAPK inhibition also results in a decrease in their transcription. IL1β gene also appears to be more transcribed in response to PLC and PKC activation which was not the case in heterophils (Table 7 vs. Table 5, respectively). In addition, PLC and PKC did not regulate IFNα, IFNγ, IL8, IL13, iNOS, TLR2, and TLR4 transcription in monocytes (Supplemental Table 3), similarly to the situation observed in heterophils (Supplemental Table 2). Interestingly, 4 h after adding the ulvan extract (25 μg/ml), PI3K acts as a transcriptional activator for IL1β and IL8, but a repressor for iNOS, TLR2, and TLR4 (Table 7). Moreover, in our conditions, only NF-κB raised iNOS, TLR2, and TLR4 transcription: its inhibition indeed results in fold changes that were at least two fold lower than the ones without inhibitor (Table 7). As for heterophils, combinatory inhibition experiments were performed. Blocking NF-κB in addition to TLR2 or TLR4 severely hampered the transcription of all the studied genes, as evidenced by qPCR (Table 8). As observed using ERK inhibitor alone, no variation of the transcription rate of the IFNα gene could be detected (Table 8).

**Table 6:** Ulvan triggers cytokine transcription in monocytes.

	**Time**	**No addition**	**Glycogen 10μg/ml**	**Ulvan 10μg/ml**	**Ulvan 25μg/ml**	**Ulvan 50μg/ml**	**Statistical difference between t = 2h and t = 4h**
**FOLD CHANGES, MONOCYTES**							
IL1β	2h	1.00 ± 0.07	1.03 ± 0.08	1.20 ± 0.09	1.15 ± 0.09	1.26 ± 0.08	p < 0.05 for ulvan 10μg/ml and 25μg/ml, p < 0.01 for 25μg/ml and 50 μg/ml
	4h	0.92 ± 0.09	0.97 ± 0.07^a^	1.63 ± 0.21^b^	5.61 ± 0.48^c*^	50.95 ± 3.72^#^
IFNα	2h	0.99 ± 0.08	1.01 ± 0.08	1.13 ± 0.09	1.16 ± 0.11	1.51 ± 0.09	p < 0.05 for ulvan 25μg/ml and p < 0.005 for 50 μg/ml
	4h	1.00 ± 0.09	1.00 ± 0.09^a^	2.58 ± 0.16^b*^	21.41 ± 0.96^#^	145.08 ± 12.69^&^
IFNγ	2h	1.02 ± 0.09	0.98 ± 0.08^a^	2.70 ± 0.012^b^	4.07 ± 0.19^c^	7.69 ± 0.25^d^	p < 0.05 for ulvan 25μg/ml and p < 0.005 for 50 μg/ml
	4h	1.02 ± 0.08	0.97 ± 0.10^a^	2.97 ± 0.21^b*^	17.61 ± 1.53^#^	147.51 ± 12.69^&^
IL8	2h	1.00 ± 0.10	0.98 ± 0.09	1.37 ± 0.12^a^	2.74 ± 0.16^b^	4.05 ± 0.33^c^	none
	4h	1.03 ± 0.09	0.09 ± 0.10	1.02 ± 0.11^a^	1.92 ± 0.15^b^	3.51 ± 0.28^c^
IL13	2h	1.02 ± 0.08	0.97 ± 0.09	1.07 ± 0.09	1.30 ± 0.11^a^	2.72 ± 0.18^b^	p < 0.05 for ulvan 10μg/ml and 25μg/ml and p < 0.01 for 50 μg/ml
	4h	1.00 ± 0.09	1.17 ± 0.16^a^	2.48 ± 0.17^b^	5.46 ± 0.51^c*^	19.01 ± 1.41^#^
TLR2	2h	0.99 ± 0.09	0.96 ± 0.10^a^	1.56 ± 0.13^b^	1.81 ± 0.17^c^	3.55 ± 0.25^d^	p < 0.05 for ulvan 25μg/ml and p < 0.01 for 50 μg/ml
	4h	1.00 ± 0.09	0.98 ± 0.09^a^	1.37 ± 0.10^b^	6.60 ± 0.52^c^	26.72 ± 1.94^d^
TLR4	2h	0.97 ± 0.08	1.00 ± 0.09	1.44 ± 0.12^a^	2.26 ± 0.21^b^	4.59 ± 0.33^c^	p < 0.05 for ulvan 10μg/ml and 25μg/ml and p < 0.01 for 50 μg/ml
	4h	0.99 ± 0.09	1.10 ± 0.17^a^	1.93 ± 1.14^b^	6.60 ± 0.52^c*^	26.72 ± 1.96#
iNOS	2h	1.00 ± 0.10	0.09 ± 0.09	1.34 ± 0.10^a^	3.52 ± 0.35^b*^	19.89 ± 2.05^#^	p < 0.05 for ulvan 10μg/ml, p < 0.01 for 25μg/ml and p < 0.005 for 50 μg/ml
	4h	0.97 ± 0.10	0.98 ± 0.09^a^	4.47 ± 0.27^b^	30.64 ± 2.16^c#^	259.62 ± 11.59^&^

*Variations are expressed as mRNA fold changes using β-actin as an internal standard. Transcription is time- and dose-dependent. Data represents the mean ± SEM of five independent experiments in triplicate. Different letters indicate statistically significant differences (a, b, c, and d) with p < 0.05 for the same incubation time, different symbols mean statistically significant differences with p < 0.01 for the same incubation time*.

**Table 7:** Transcription in monocytes relies on TLR2 and TLR4 activation and requires intra-cellular mediators that differ according to the genes.

**Inhibitor target**	**IL1β**	**IFNα**	**IFNγ**	**IL8**	**IL13**	**TLR2**	**TLR4**	**iNOS**
**Fold changes, monocytes**								
No inhibitor	24.93 ± 2.43	42.96 ± 3.21	64.54 ± 5.01	12.09 ± 1.43	4.25 ± 0.50	12.39 ± 0.93	29.84 ± 2.18	53.59 ± 4.69
TLR2	12.24 ± 1.12^a^	15.14 ± 1.53^b^	28.89 ± 0.84^a^	4.01 ± 0.38^a^	2.14 ± 0.12^a^	5.41 ± 0.27^a^	15.62 ± 1.15^a^	27.34 ± 2.23^a^
TLR4	10.43 ± 0.97^a^	11.79 ± 1.54^b^	26.32 ± 0.62^a^	5.04 ± 0.55^a^	1.78 ± 0.12^a^	8.69 ± 0.17^a^	11.40 ± 1.01^a^	25.58 ± 2.40^a^
TLR2+4	1.16 ± 1.28^c^	2.08 ± 0.19^c^	1.09 ± 0.09^c^	0.99 ± 0.08^c^	1.21 ± 0.11^a^	1.08 ± 0.10^b^	1.16 ± 0.11^c^	2.89 ± 0.30^c^
TLR4+21	4.74 ± 0.35^b^	11.79 ± 0.10^b^	24.30 ± 0.71^a^	5.74 ± 0.32^a^	1.88 ± 0.19^a^	7.78 ± 0.21^a^	12.51 ± 0.14^a^	25.87 ± 2.41^a^
TLR9	20.13 ± 2.08	39.58 ± 2.85	58.44 ± 4.45	11.95 ± 1.53	4.23 ± 0.07	11.62 ± 0.84	25.09 ± 2.31	55.74 ± 2.30
Cytoplasmic TLR	25.05 ± 3.69	41.88 ± 3.56	68.33 ± 6.04	11.78 ± 1.43	4.33 ± 0.19	12.36 ± 0.86	27.92 ± 2.47	54.58 ± 3.08
NOD	26.57 ± 0.70	43.27 ± 2.27	67.48 ± 6.12	12.45 ± 1.47	4.11 ± 0.08	12.08 ± 0.99	29.67 ± 3.23	50.56 ± 3.95
Dectin	24.49 ± 1.41	44.31 ± 2.36	66.87 ± 5.97	12.35 ± 1.74	4.13 ± 0.32	11.93 ± 1.02	30.55 ± 3.61	54.77 ± 4.38
NLRP1+3	24.73 ± 1.81	42.24 ± 2.27	67.30 ± 6.66	1.81 ± 0.96	4.24 ± 0.15	11.59 ± 0.99	29.67 ± 3.01	54.39 ± 4.70
NLRP3	25.31 ± 1.82	41.91 ± 1.36	64.07 ± 5.85	12.52 ± 1.60	4.48 ± 0.24	12.16 ± 1.05	27.78 ± 2.67	52.99 ± 3.36
NF-KB	3.66 ± 0.32^b^	5.96 ± 0.55^c^	5.70 ± 0.52^c^	2.88 ± 0.23^a^	2.49 ± 0.19^a^	5.34 ± 0.30^a^	4.43 ± 0.36^a^	8.92 ± 0.82^c^
ERK	6.34 ± 0.59^b^	44.86 ± 4.81	44.61 ± 4.22^a^	4.26 ± 0.38^a^	4.12 ± 0.42	12.38 ± 1.12	32.51 ± 2.85	51.75 ± 3.85
JNK	11.14 ± 1.12^a^	44.12 ± 4.34	66.85 ± 5.86	4.17 ± 0.47^a^	4.05 ± 0.37	11.72 ± 1.06	30.70 ± 2.72	55.49 ± 3.95
p38 MAPK	4.02 ± 0.35^b^	43.36 ± 3.98	64.97 ± 4.79	4.58 ± 0.34^a^	4.55 ± 0.31	13.03 ± 1.23	29.95 ± 2.62	57.23 ± 4.21
PLC	14.71 ± 1.13^a^	43.87 ± 4.08	68.40 ± 5.95	11.50 ± 1.06	4.23 ± 0.41	12.85 ± 1.25	30.69 ± 2.96	54.75 ± 5.22
PKC	13.1 ± 1.11^a^	41.42 ± 4.12	65.92 ± 5.19	11.55 ± 1.14	4.12 ± 0.39	12.69 ± 1.30	31.33 ± 3.12	53.74 ± 5.29
PI3K	13.41 ± 1.24^a^	41.15 ± 3.75	65.73 ± 6.23	3.67 ± 0.19^a^	4.23 ± 0.43	18.74 ± 1.82^a^	57.72 ± 4.16^b^	117.73 ± 9.86^c^

*For the inhibition experiments, monocytes were first incubated for 30 min with inhibitors specific for different PRRs' or intracellular proteins before addition of the ulvan extract at 25 μg/ml for 4 h. Data represents the mean ± SEM of five independent experiments in duplicate. Different letters indicate statistically significant differences, ^a^p < 0.05, ^b^p < 0.01, ^c^p < 0.05*.

**Table 8:** TLR2 and TLR4 pathways regulate transcription in monocytes through common intracytoplasmic mediators.

**Inhibitor target**	**IL1β**	**IFNα**	**IFNγ**	**IL8**	**IL13**	**TLR2**	**TLR4**	**iNOS**
**FOLD CHANGES, MONOCYTES**								
No inhibitor	25.39 ± 0.66	43.40 ± 2.18	66.39 ± 2.67	11.90 ± 1.09	4.41 ± 0.39	12.22 ± 1.13	29.45 ± 2.28	55.72 ± 4.63
TLR2	12.22 ± 1.58	15.06 ± 1.48	27.39 ± 1.41	4.48 ± 0.23	2.16 ± 0.16	5.98 ± 0.52	15.14 ± 1.48	26.14 ± 2.13
TLR2 + NFKB	1.41 ± 0.24^b^	0.88 ± 0.21^b^	1.83 ± 0.38^b^	1.19 ± 0.21^a^	0.90 ± 0.10^a^	0.87 ± 0.09^a^	1.04 ± 0.11^b^	1.03 ± 0.10^b^
TLR2 + ERK	3.25 ± 0.31^b^	12.97 ± 1.97	9.36 ± 0.77^a^	1.43 ± 0.11^a^	2.04 ± 0.018	2.63 ± 0.24	13.21 ± 1.19	22.68 ± 2.53
TLR2 + JNK	5.24 ± 0.41^a^	13.69 ± 0.96	27.46 ± 2.59	1.78 ± 0.13^a^	2.04 ± 0.17	3.14 ± 0.25	15.38 ± 1.49	26.15 ± 2.05
TLR2 + p38	1.87 ± 0.15^b^	13.00 ± 1.41	24.93 ± 1.57	1.63 ± 0.14^a^	2.00 ± 0.11	3.08 ± 0.29	14.73 ± 1.31	25.53 ± 2.24
TLR2 + PLC	5.66 ± 0.34^a^	14.63 ± 2.72	27.80 ± 2.29	4.39 ± 0.46	2.03 ± 0.20	3.24 ± 0.23	14.33 ± 1.07	24.09 ± 2.19
TLR2 + PKC	4.74 ± 0.30^a^	12.49 ± 1.18	26.58 ± 2.64	4.49 ± 0.42	2.11 ± 0.18	3.24 ± 0.31	14.91 ± 1.44	25.95 ± 1.99
TLR2 + PI3K	3.19 ± 1.20^b^	13.09 ± 1.54	28.13 ± 1.83	2.77 ± 0.10^a^	2.14 ± 0.21	10.76 ± 1.03^a^	30.82 ± 2.96^a^	48.23 ± 4.29^a^
TLR4	10.44 ± 1.50	11.123 ± 1.17	21.99 ± 2.04	5.55 ± 0.48	1.85 ± 0.13	8.09 ± 0.88	15.99 ± 1.48	5.561 ± 5.26
TLR4 + NFKB	1.73 ± 0.14^b^	0.94 ± 0.18^b^	2.32 ± 0.52^b^	1.91 ± 0.10^a^	0.92 ± 0.08^a^	1.68 ± 1.14^b^	1.06 ± 0.10^b^	0.98 ± 0.11^b^
TLR4 + ERK	5.85 ± 0.54^a^	11.60 ± 1.08	14.45 ± 1.48^a^	2.29 ± 0.25^a^	1.73 ± 0.14	10.94 ± 1.05	15.64 ± 1.41	5.66 ± 0.52
TLR4 + JNK	7.60 ± 0.72^a^	11.99 ± 2.03	21.21 ± 2.19	2.11 ± 0.23^a^	1.78 ± 0.15	10.88 ± 1.01	15.30 ± 1.49	5.30 ± 0.52
TLR4 + p38	1.68 ± 0.13^b^	12.42 ± 1.57	7.12 ± 0.68	2.31 ± 0.21^a^	1.78 ± 0.12	11.27 ± 1.12	14.06 ± 1.33	5.75 ± 0.51
TLR4 + PLC	7.34 ± 0.72^a^	10.98 ± 1.06	22.88 ± 2.34	5.51 ± 0.59	1.78 ± 0.16	10.68 ± 1.02	14.84 ± 1.41	5.84 ± 0.57
TLR4 + PKC	7.47 ± 0.74^a^	10.85 ± 1.09	20.78 ± 2.56	5.30 ± 0.48	1.79 ± 0.13	9.76 ± 0.95	15.71 ± 1.47	5.78 ± 5.57
TLR4 + PI3K	6.99 ± 0.69^a^	10.76 ± 1.07	20.06 ± 1.95^a^	2.24 ± 0.21^a^	1.76 ± 0.16	18.74 ± 1.32^a^	28.815 ± 2.85^a^	10.670 ± 1.57^a^

*Variations are expressed as mRNA fold changes using β-actin as an internal standard. Monocytes were first incubated for 30 min with the inhibitors before addition of the ulvan extract at 25 μg/ml for 4 h. Data represents the mean ± SEM of four independent experiments in triplicate. Letters indicate statistically significant differences with the experiment with the TLR inhibitor, ^a^p < 0.05, ^b^p < 0.01*.

### Ulvan also acts *in vivo* when given *per os*

To address the potential biological effects of ulvan *in vivo*, three independent experiments were performed, each with four groups of 25 chickens given ulvan at 0, 10, 25, 50 mg/l in drinking water during 24 h. Heterophils and monocytes were purified each day from day 0 to day 3, and plasmas kept frozen. No loss of appetite, aggressivity, wounding, abscess, fever or mortality was observed at any time by the veterinary assessment performed each day for all the repetitions. The animals had an exploratory behavior with no huddled chickens. Weight were similar between groups and between experiments and correspond to the standards established by the genetic company (Supplemental Table 4).

**Figure 4:**
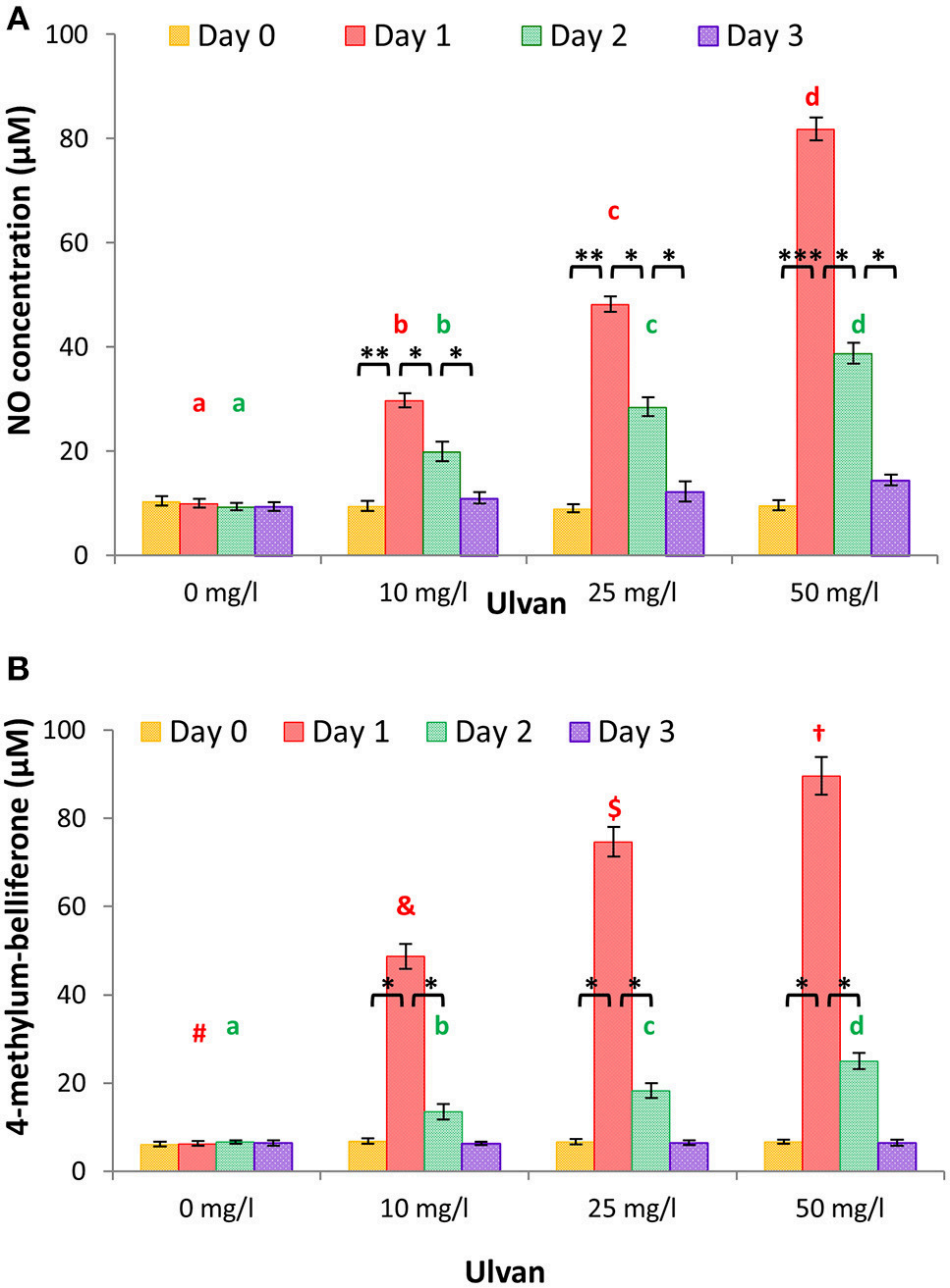
Ulvan acts *in vivo* when given *per os*. Ulvan was given *per os* at four different concentrations (0, 10, 25, 50 mg/l) to the animals at day 0. Blood samples (1ml per animal) were taken every day from day 0 to day 3. The NO concentration and β-D-glucuronidase activity were quantified individually in the plasma, the latter via the 4-methylum-belliferone formation. A dose response release is observed for both NO **(A)** and β-D-glucuronidase **(B)** with a peak at day 1. Data represents the mean ± SEM of three independent experiments with 25 chickens per group. Different letters with the same color indicate statistically different values for the different doses at the same time with *p* < 0.05, different symbols with the same color indicate statistically different values for the different doses at the same time with *p* < 0.01. *p < 0.05, **p < 0.01, and ***p < 0.005 when values are statistically different for the same dose at different times.

The NO concentration was quantified in plasma from individual chickens as described above, since it mirrors, at least in part, monocyte activation. A dose- and time-dependent release was observed with maximal concentrations at day 1 with values of 9.99 ± 0.85 μM for the negative control, 29.72 ± 1.34 μM with 10 mg/l ulvan, 48.22 ± 1.51 μM with 25 mg/l ulvan, and 81.8 ± 6.50 μM with 50 mg/l ulvan (Figure 4A). The groups without ulvan stimulation did not present any statistically significant variations over time, with NO concentrations between 10.41 ± 0.71 μM and 9.34 ± 0.35 μM.

Release of granules by heterophils, and potentially by monocytes, was measured by quantification of the amount of β-D-glucuronidase activity. As shown in Figure 4B, increased ulvan concentrations led to higher 4-methylum-belliferone concentrations, reflecting a greater subset of heterophils and/or monocytes being activated. All the series displayed a peak of β-D-glucuronidase activity at day 1, swiftly declining, with values no longer statistically different from that of the negative control at day 3. At day 1, β-D-glucuronidase in the plasmas allowed 4-methylum-belliferone concentrations to rise to 6.29 ± 0.48 μM for the negative control, 48.7 ± 2.85 μM with 10 mg/l ulvan, 74.67 ± 3.38 μM with 25 mg/l ulvan, 89.61+/4.23 μM with 50 mg/l ulvan. In the groups without any ulvan added, no variation was observed with 4-methylum-belliferone concentrations ranging from 6.12 ± 0.55 μM to 6.63 ± 0.39 μM.

### Both heterophils and monocytes respond to ulvan *in vivo*

In order to gain insight into the mechanisms of action of ulvan, RT-qPCR analyses were carried out on monocytes and heterophils purified from blood samples taken at 0, 24, 48, and 72 h after ulvan administration. Since blood samples were only 1 ml per animal, samples were first centrifuged to separate the plasma from the cells. The cellular pellets of three animals were then pooled to obtain sufficient amount of cells for purification.

**Figure 5:**
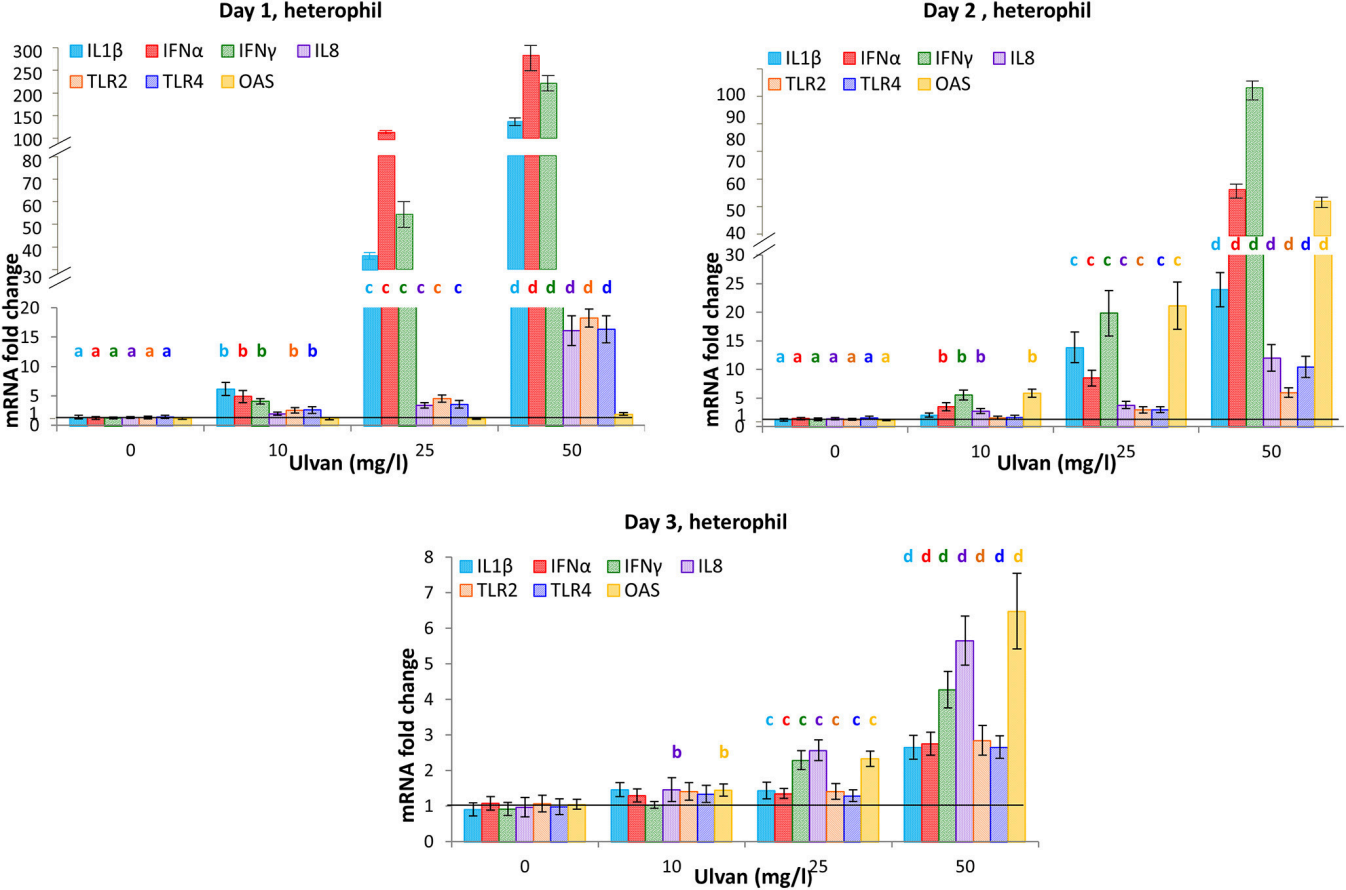
Heterophils respond to ulvan by modifying the transcription pattern of cytokine genes. Ulvan was given *per os* at four different concentrations (0, 10, 25, 50 mg/l) to the animals at day 0. Blood samples (1 ml per animal) were taken every day from day 0 to day 3. Cellular pellets of three animals were pooled to obtain sufficient number of cells for purification and RT-qPCR. Data represents the mean ± SEM of three independent experiments with 25 chickens per group. Different letters with the same color indicate statistically different values for the different doses at the same time with *p* < 0.05.

Heterophils responded as early as day 1 by tuning on the transcription of the pro-inflammatory genes for IL1β, IFNα, IFNγ, and to a lower extent those of IL8, TLR2, and TLR4 (Figure 5). Fold changes were dose-dependent and varied from 6.17± 1.12 with 10 mg/l ulvan to 137.68 ± 16.20 with 50 mg/l ulvan for IL1β, from 4.90 ± 1.04 with 10 mg/l ulvan to 290.14 ± 46.86 with 50 mg/l ulvan for IFNα, from 4.08 ± 0.46 with 10 mg/l ulvan to 225.82 ± 22.15 with 50 mg/l ulvan for IFNγ. Fold changes decreased from day 2 for these genes but remained statistically different at day 2 (Figure 5). TLR2 and TLR4 were also more transcribed with ulvan, although in a lesser extent than the previous genes. IL8 displayed a different transcription pattern with only moderate fold changes at day 1 (1.96 ± 0.30 with 10 mg/l ulvan to 16.13 ± 2.57 with 50 mg/l ulvan), that did not significantly decrease at day 2 (2.73 ± 0.41 with 10 mg/l ulvan to 11.99 ± 2.32 with 50 mg/l ulvan) but decreased at day 3 (Figure 5). Thus, induction of its transcription appears to be less acute than those of IL1β, IFNα, IFNγ, but to last longer than the one of IL1β for the highest doses (25 and 50 mg/l). The transcription of 2′-5′ Oligoadenylate synthase (OAS) was also delayed when compared with those of IL1β, IFNα, IFNγ. Fold changes were indeed identical to the ones of the control at day 1, maximal at day 2 ranging from 5.84 ± 0.71 with 10 mg/l ulvan to 51.63 ± 8.9 with 50 mg/l ulvan and remaining statistically increased compared to the controls at day 3 for the highest concentrations, albeit to a lesser extent (Figure 5). All the other cytokines genes analyzed (IL10, IL13, IL17, IL18, IFNβ) were not significantly tuned on nor tuned off during the experiments (Supplemental Table 5). Moreover no change was observed in the Ct and ΔCt values for the series without ulvan from day 0 to day 3; they also did not differ from the levels observed in the *in vitro* experiments (Supplemental Table 6).

**Figure 6:**
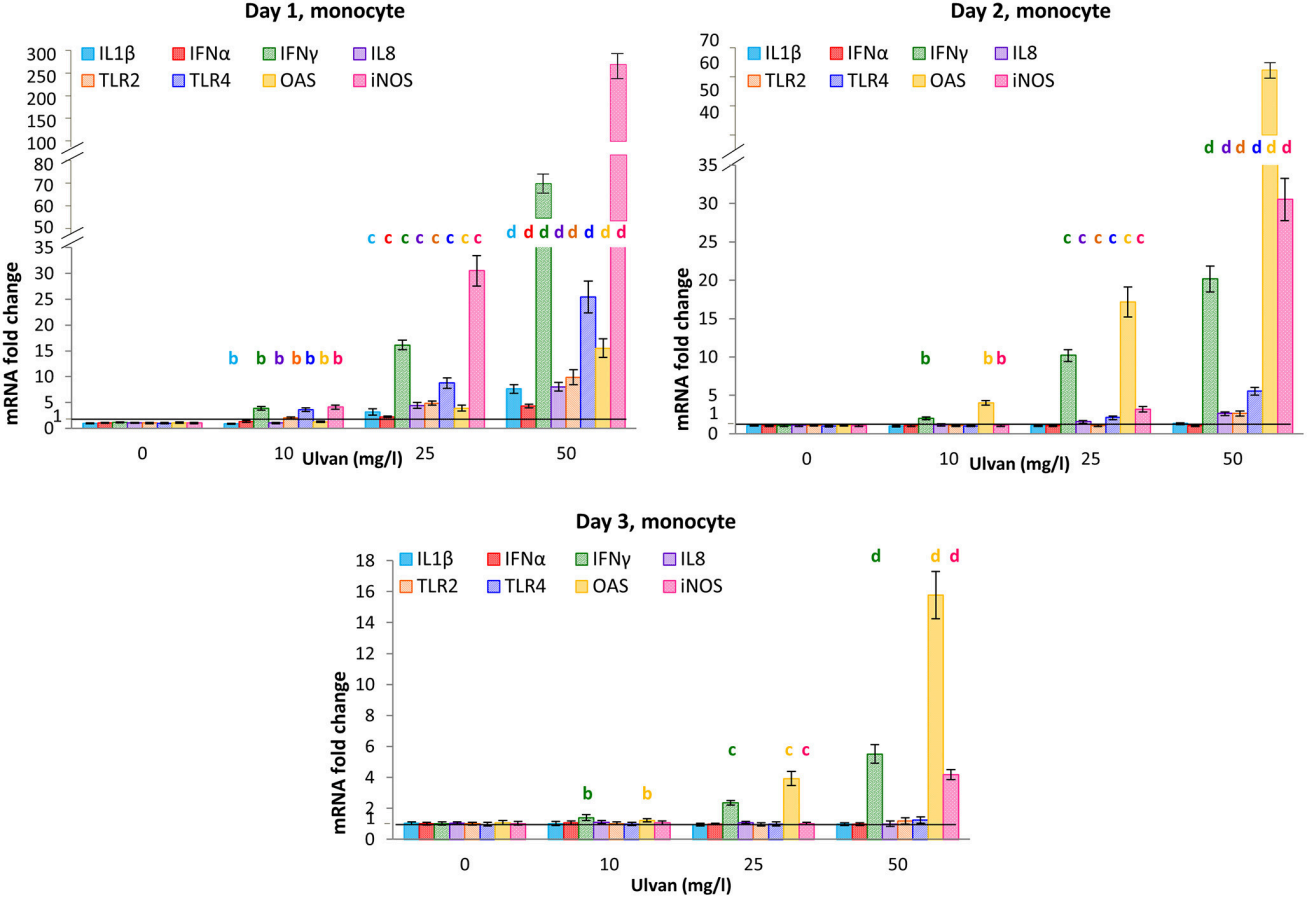
Cytokines mRNA are transcribed in monocytes in response to ulvan. Ulvan was given *per os* at four different concentrations (0, 10, 25, 50 mg/l) to the animals at day 0. Blood samples (1 ml per animal) were taken from day 0 to day 3. Cellular pellets of three animals were pooled to obtain sufficient number of cells for purification and RT-qPCR. Data represents the mean ± SEM of three independent experiments with 25 chickens per group. Different letters with the same color indicate statistically different values for the different doses at the same time with *p* < 0.05.

The transcription patterns observed for the monocytes appeared to differ somewhat from the ones for heterophils. Common to both cellular types is the fact that the acute phase of the response was detected at day 1. However, despite increased fold changes, the transcription of IL1β gene was less affected in monocytes than in heterophils with values at day 1 ranging only from 0.89 ± 0.08 with 10 mg/l ulvan to 7.63 ± 0.82 with 50 mg/l ulvan (Figure 6). As a result and contrarily to what was observed for heterophils (Figure 6), fold changes returned to around 1 as early as day 2 (Figure 6). Similarly, transcription of IL8 and IFNα genes was activated in a significant manner in monocytes, but less than in heterophils. For instance, the fold change for IFNα at day 1 with 50 mg/l ulvan rose to 4.31± 0.37 in monocytes (Figure 6) while it was 290.14 ± 46.86 in heterophils (Figure 5). Transcription of the IFNγ gene was also increased in a dose-dependent manner at day 1, with fold changes that rose to 3.88 ± 0.37 with 10 mg/l ulvan and 66.72 ± 5.95 for 50 mg/l ulvan (Figure 6). Once again the fold changes at day 1 in monocytes were lower than in heterophils, but the tendency between heterophils and monocytes was similar, with an about 2-fold decrease between day 1 and day 2, and quite similar fold changes at day 3. The gene which transcription was severely raised in monocytes, especially at day 1, is the iNOS one, with a transcription pattern reminiscent of the IFNγ one. At this time a dose-dependent response occurred with fold changes ranging from 3.88 ± 0.37 with 10 mg/l ulvan to 258.25 ± 23.25 with 50 mg/l ulvan (Figure 6). However increased transcription appears to be transient as fold changes decreased with a factor nearly eight at day 2, and for instance a fold change that rose only to 30.51 ± 4.14 with 50 mg/l ulvan at day 2. Moreover at day 3 the values were equal to one, except for the highest concentration with a fold change of 4.18 ± 0.32, which is yet about 60 times less than the one at day 1 (268.25 ± 23.25). Otherwise, as observed in heterophils, the transcription was increased for OAS. However, a significant increase appeared as early as day 1 in monocytes with fold changes equal to 3.90 ± 0.59 and 15.53 ± 1.83 with 25 mg/l and 50 mg/l ulvan, respectively (Figure 6) while variations were not observed before day 2 for heterophils (Figure 6). As in heterophils the peak of transcription was day 2 in monocytes with values equal to 4.01 ± 0.30 with 10 mg/l and 62.76 ± 5.08 with 50 mg/l ulvan before decreasing at day 3 to reach 1 except for the highest concentrations (Figure 6). The transcription of all the other cytokines genes analyzed (IL10, IL13, IL17, IL18, IFNβ) did not vary significantly during the experiments (Supplemental Table 7). Moreover no change was observed in the Ct and ΔCt values for the series without ulvan from day 0 to day 3; they also did not differ from those observed in the *in vitro* experiments (Supplemental Table 8).

### Cytokines are secreted *in vivo* in a context of moderate and transient inflammation

**Figure 7:**
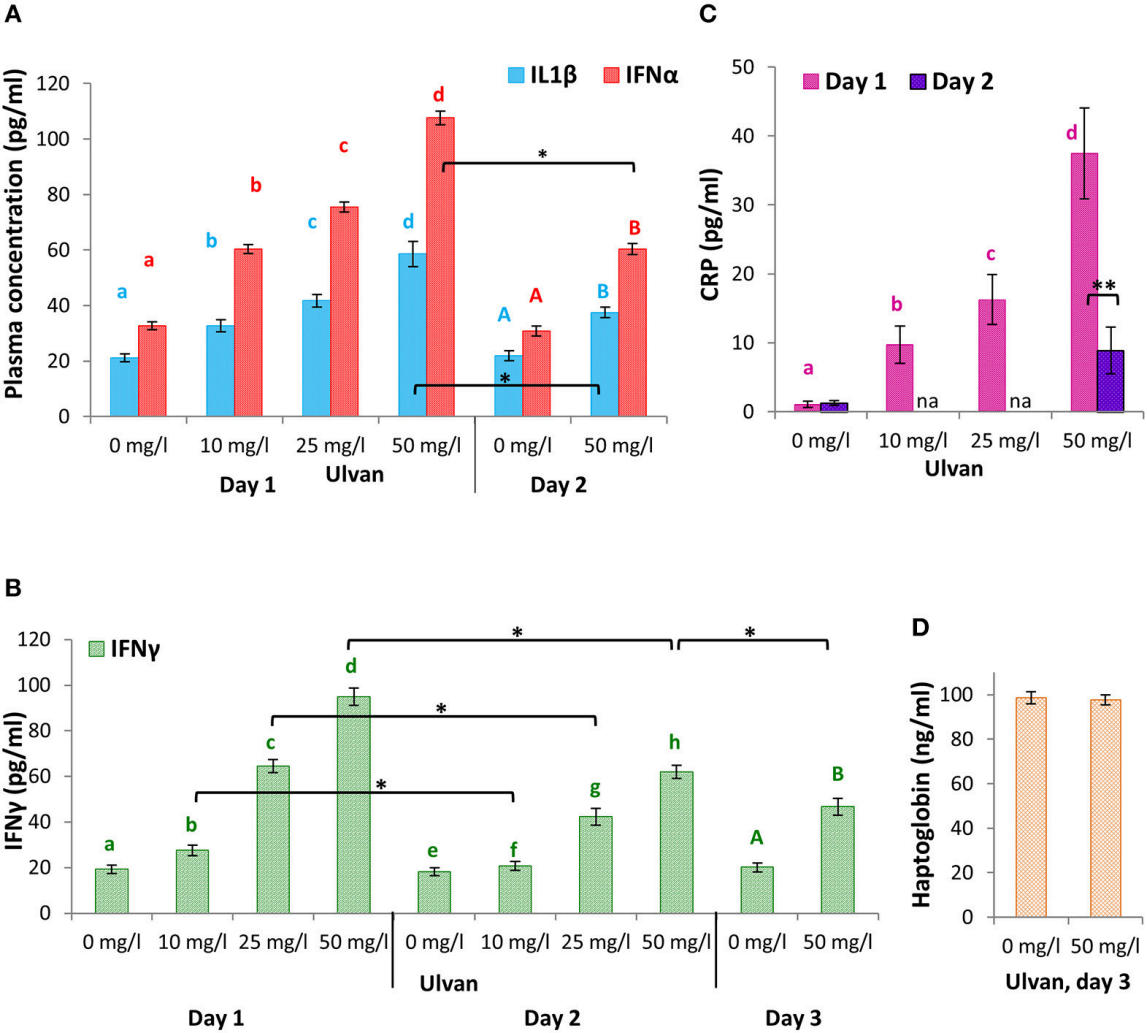
IL1β, IFNα, and IFNγ are released in a context of transient and moderate inflammation context. Ulvan was given *per os* at four different concentrations (0, 10, 25, 50 mg/l) to the animals at day 0. Blood samples (1 ml per animal) were taken every day from day 0 to day 3. ELISA were performed on each plasma to quantify the concentrations of IL1β and IFNα **(A)**, IFNγ **(B)**, C-Reactive Protein **(C)**, and haptoglobin **(D)**. Data represents the mean ± SEM of three independent experiments with 25 chickens per group. Different letters with the same color indicate statistically different values for the different doses at the same time with *p* < 0.05. *p < 0.05, when values are statistically different for the same dose at different times.

To address whether the variations in mRNA amounts correlated with cytokines release, ELISA assays were performed for IL1β, IFNα, and IFNγ (Figure 7). IL1β concentrations rose in a dose-dependent manner as early as day 1 from 21.17 ± 0.96 pg/ml without ulvan supplementation, to 32.65 ± 1.15 pg/ml with 10 mg/l ulvan and 58.56 ± 1.06 pg/ml with 50 mg/l ulvan, in agreement with the increased fold changes observed in heterophils and monocytes. Secretion appeared to be transient as a statistically significant one third decrease occurred in a single day so that 50 mg/l ulvan resulted in a plasmatic concentration of 37.53 ± 1.43 pg/ml at day 2, as compared to 21.84 ± 1.81 pg/ml without any ulvan stimulation on the same day (Figure 7A). The IFNα secretion pattern was similar to that of IL1β, with a dose-dependent response at day 1 decreasing in a statistically significant manner at day 2. Concentrations at day 1 were however higher than those of IL1β and ranged from 32.66 ± 2.81 pg/ml without ulvan supplementation to 60.35 ± 3.33 pg/ml with 10 mg/ml and 107.63 ± 7.49 pg/ml with 50 mg/l ulvan. This was also true at day 2 with a nearly unchanged value for the untreated group (30.80 ± 2.74 pg/ml) and a value of 60.28 ± 5.96 pg/ml for the highest ulvan dose (Figure 7A). Once more these concentrations were in accordance with the fold change evolution observed in monocytes and heterophils over time. It can thus be suggested that the two cellular types may contribute to release of IL1β. IFNγ concentration in plasmas was 19.33 ± 1.82 pg/ml without ulvan supplementation, and rose to 27.57 ± 2.25 pg/ml with 10 mg/l ulvan and to 94.95 ± 3.81 pg/ml with 50 mg/l ulvan. Despite being statistically significant, the decline in concentrations at day 2 was nevertheless less acute than for IL1β and IFNα (Figure 7B). Consequently, IFNγ concentration at day 3 remained more than twice the one found without ulvan supplementation (46.69 ± 3.75 pg/ml vs. 20.14 ± 1.94 pg/ml with 50 mg/l ulvan, Figure 7B). No statistical difference was observed for IL1β, IFNα, and IFNγ concentrations for the control group in any of the three repetitions and over the 4 days (from day 0 to day 3).

As IFNα and IFNγ may bridge over other cellular populations for activation, and as autocrine and/or paracrine loops may occur, we assessed the extent of the inflammation initiated by the ulvan, at an early stage by quantifying C - Reactive Protein (CRP), and at a later stage by measuring haptoglobin. CRP was synthetized by the liver in response to ulvan in a dose-dependent manner. The plasmastic CRP concentrations reached 1.08 ± 0.06 ng/ml without ulvan supplementation and rose at day 1 to 9.74 ± 0.36 ng/ml with 10 mg/l ulvan and 37.49 ± 0.85 ng/ml with 50 mg/l ulvan (Figure 7C). Day 2 was characterized by CRP concentration falling to 8.91 ± 0.55 ng/ml with 50 mg/ml ulvan, while remaining constant for the untreated group (1.27 ± 0.08 ng/ml). Concentrations for haptoglobin at day 3 were nearly identical between the control group and the group with 50 mg/l ulvan, 98.59 ± 4.72 vs. 97.66 ± 6.16, respectively (Figure 7D).

## Discussion

The immune system is constantly exposed to a large variety of threatening and potentially damaging agents and uses complex cellular and molecular mechanisms to determine the appropriate response to each situation: Whether to activate the adaptive immunity or if the innate immune response may be sufficient. The latter one involves different populations of mononuclear cells (monocytes, macrophages, NK, NKT, B, and γδ T lymphocytes) and polynuclear cells.

The primary polymorphonuclear leukocyte in chicken is the heterophil. It provides a rapid deployment of the effector arm of the innate immune system in birds, displaying a variety of pathogen recognition receptors, including toll-like receptors (TLRs), which account for the recognition of a multitude of pathogens. The TLR family in chickens consists of ten genes, where TLR2 and TLR4 are orthologs to mammal TLRs (20).

In our experiments, heterophils constitutively express TLR2 and TLR4, as previously reported (21). Kogut et al. demonstrated that activation of heterophils with TLR2 and TLR4 with agonists, respectively PAM and LPS, results in an oxidative burst and degranulation (21, 22). In our hands, when using the same technologies the biological effects obtained were close to the ones previously described, when in the same conditions (21, 22). Moreover, thanks to the use of specific inhibitors, we have demonstrated that ulvan activates TLR2 and TLR4 on heterophils *in vitro*. Ulvan thus appears as a new biological ligand for chTLR2 and chTLR4 (chicken TLR2 and TLR4), in addition to the ones previously described (4, 12).

As human TLR4 has been described to bind palmitic acid (1, 4) and as no data are currently available for chTLR, we quantified the amount of palmitic acid both in the extract and in the feed (Supplemental Figure 2). As the feed intake for chicken at 25 days of age is 130 g of pelleted feed per day per animal, the daily intake of palmitic acid via the feed was calculated to 538 mg per day. As they also consume 200 ml of water, the chickens with the highest concentration of ulvan extract received an additional load of 0.6 μg per animal during the first day of the experiment. Thus the extra palmitate amount brought by the highest concentration of the ulvan extract represents 1.12 × 10^−6^ fold less than the amount of the palmitate owing to the pelleted feed. As we also failed to detect any endotoxin (TLR ligand), we considered that the biological effects observed were achieved thanks to the ulvan. However, *in vitro*, ulvan seems to be a less potent activator of the oxidative burst than LPS, the TLR4 agonist, as about 1.5-fold more ulvan than LPS was required to reach similar relative fluorescent units. This was also observed for TLR2, where a five times higher concentration of ulvan than PAM, the TLR2 agonist, was required to reach the same biological effects.

Results for the degranulation are consistent with the ones for the burst, thus confirming that ulvan is a less potent activator of chTLR2 and chTLR4 than PAM and LPS *in vitro*. In addition, we have established, for the first time to our knowledge, that chTLR2 and chTLR4 cooperate to control avian heterophil activation, as evidenced for the oxidative burst and for the degranulation. Nevertheless, Keestra et al. reported that chTLR2 and chTLR4 are present as membrane receptors and that homodimers are required for signal transduction (4). In this study, we cannot state precisely whether the receptors dimers activated by ulvan are the homozygous ones and/or hypothetical heterologous ones (TLR2/TLR4) as the presence of the last ones (TLR2/TLR4) cannot be formally ruled out. Furthermore, since the extract may contain chains differing in their composition and size, we cannot exclude that different dimers may be activated at the same time by several ulvan subtypes.

When analyzing the transduction pathways, neither the burst nor the degranulation were statistically significantly affected by the specific inhibitors against p38MAPK, JNK, ERK, NF-κB, as previously described (13, 16). Moreover, we have also evidenced for the first time to our knowledge, that PI3K is involved in the degranulation process while it may not be required for the oxidative burst, as its inhibition did not result in a statistically significant decrease of the fluorescence for the burst (*p* = 0.056).

Two proteins appear as the main regulators of both degranulation and oxidative burst, PKC and PLC. Interestingly, for the burst, they both seem to act simultaneously on chTLR4 and chTLR2 transduction pathways and with the same efficiency (Table 2). PKC is also required for chTLR2 and chTLR4 dependent degranulation (Table 2). ChTLR2 dependent degranulation also implies PLC, as blocking chTLR4 and PLC together such as only chTLR2 can be activated, resulted in a 4-methylumbelliferone release that was statistically lower than when only chTLR4 was blocked (*p* < 0.05, Table 2). However, this synergy was not observed when simultaneously inhibiting TLR2 and PLC (Table 2), thus indicating that chTLR4 dependent degranulation may not require PLC while chTLR2 dependent one does. All in all, chTLR2 activation with ulvan results in a degranulation and an oxidative burst that are both supported by PKC and PLC activation, while chTLR4 activation leads to a degranulation process relying on PKC but not PLC, and an oxidative burst requiring PKC and PLC.

Due to the lack of myeloperoxidase, avian heterophils produce only weak amounts of NO and no Neutrophil Extracellular Trap as myeloperoxidase is required for this release (18, 23). Therefore NO quantification was not performed for the supernatants of heterophils incubated with ulvan but only for those of monocytes. In this model ulvan induces a dose- and time-dependent NO release. It relies on TLR2 and TLR4 activation with a synergic effect and requires about five fold more ulvan than PAM or LPS, the respective TLR2 and TLR4 agonists. However, the NO concentrations (no more than 25 μM) were lower than the ones observed by Barjesteh et al. on the chicken MQ-NCSU macrophage cell line [around 100 μM, (24)]. This discrepancy may be accounted for by the difference in the cellular type, the genetic background (Ross vs. Dekalb XL) and the incubation time (48 h in Barjesteh's work as compared to 4 h in the present work).

In a second step, we examined whether chTLR2 and chTLR4 activation with ulvan may result in modifications of the transcription pattern for heterophils and monocytes. We focused on cytokines involved in the innate immune response. As previously described, we have observed that TLR stimulation on heterophils *in vitro* results in the transcription of pro-inflammatory cytokines genes, especially IL1β (21, 23). Similarly to the results of Kogut et al. in a model of heterophils highly responding to *Salmonella Enteritidis*, we also observed that chTLR2 and chTLR4 transduction pathways involve MAP kinases and NF-κB to regulate IL1β, IL8, IL18, IFNα, and IFNγ (25). However, the reason why the transcription of chTLR2 and chTLR4 genes is enhanced by NF-κB and repressed by PI3K remains to be explained (Table 3). One hypothesis might be protection mechanism with as a first step, activation of transcription by NF-κB to increase the number of membrane receptors and thus improve pathogen capture; and as a second step, PI3K involvement to avoid a metabolically expensive and unnecessary transcription, when the membrane TLR are no more activated due to their ligands' clearance (26).

This mechanism may be common to heterophils and monocytes, as the same chTLR2 and chTLR4 regulation pattern arises in monocytes. In addition, we observed that the iNOS gene is regulated in a similar manner to the TLR2 and TLR4 genes in monocytes. Our previous hypothesis may thus also apply to this gene, in order to allow a sufficient amount of NO to be released for pathogen killing, but not too excessive or too long lasting in order to avoid cellular damage to healthy cells. Our results are consistent with those of Peroval et al for the avian HD11 macrophage cell line with the same inhibitors for PI3K, (wortmaninn), NF-κB, p38MAPK and ERK (PD98059), with PAM and LPS as agonists for TLR2 and TLR4, respectively (27). They showed the iNOS gene to be induced by NF-κB and repressed by PI3K. Furthermore, and in agreement with our results, induction of IL1β, IFNα, and IFNγ genes after TLR2 or TLR4 activation has also been described by Barjesteh et al using the MQ-NCSU avian macrophage cell line (24). Interestingly this team demonstrated that activating membrane TLR2 or TLR4 on these macrophages inhibits influenza virus H4N6 replication *in vitro* and increases its shedding *in vivo* (24, 28, 29). In addition, Haddadi et al also evidenced using this cell line that induction of TLR4 signaling inhibits laryngotracheitis virus replication (30).

However, the transcriptional regulation appears to differ somewhat between heterophils and monocytes for IL1β, IL8, IL18, IFNα, and IFNγ, thus suggesting that other cell-type specific mechanisms could be involved. This would for instance explain the discrepancy of IL13 gene regulation observed between the two cellular types.

Nevertheless, in both cellular types TRL4 and TLR2 share common intracellular mediators. Within a cellular type each mediator acts in a similar extent on TLR2 and TRR4 pathways (Tables 5, 8).

Given the *in vitro* results and as chTLR activation may be of use in prophylaxis, we finally examined whether ulvan may stimulate, directly or indirectly, the chicken innate systemic immunity *in vivo*. We first measured the inflammation level to exclude any deleterious effects on the animals' health.

Plasma concentration of CRP rose in a dose dependent-manner to values that are above the normal (1.56–8.6 ng/ml, according to the manufacturer) except for the control group and the 10 mg/l ulvan group. All groups were back to normal as soon as day 2. Furthermore, haptoglobin concentrations did not vary and remained within normal range for all the groups (93–186 ng/ml, according to the manufacturer). Ulvan thus appears to induce a transient and moderate inflammation. In addition, as no change in the animals' behavior was observed for the three independent experiments (from 1 week before the experiment until slaughtering), and as Ct value and Delta Ct values of all the studied genes are extremely close between the *in vitro* experiments and the control group *in vivo*, we have considered that no undesired event occurred during the *in vivo* experiments (Supplemental Tables 6, 8).

We have evidenced, for the first time to our knowledge, that β-D-glucuronidase, a lysosomal enzyme, was released in a dose dependent manner at day 1 after ulvan oral intake. This reflects heterophil activation and potentially monocyte activation (31). In addition to β-D-glucuronidase, activated heterophils may also release anti-microbial compounds including, β-defensins, cathepsins, lysozyme, acid phosphatase α-glucuronidase and elastase, as previously described *in vitro* (18) and *in vivo* (31, 32).

In line with this result, NO was also present in plasma. Nevertheless, we cannot exclude that cellular types other than monocytes may contribute to NO synthesis, as demonstrated for instance in mammalian endothelial cells (33). In a murine model of neutrophils lacking the ability to produce Reactive Oxygen Species (as is the case for heterophils), the clearance of different size pathogens involves distinct inflammatory programs (34). This study highlights the key role of IL1β in this process, irrespective of the inflammation program, with a statistically significant release of IL1β by neutrophils, in line with our own RT-qPCR results. However, we cannot formally rule out that monocytes may also contribute to IL1β production, even if, in our experiments, the gene was less tuned on in monocytes than in heterophils. Quantification of the IL1β concentration in plasma confirms its presence at levels above the normal values (15 to 30 pg/ml, according to the manufacturer) consistent with the RT-qPCR results. In the same register, IFNα and IFNγ were also released with a peak at day 1, once again in accordance with the RT-qPCR. In both cases their concentrations were in the same range at day 1 and higher than the control, from 29 to 42 pg/ml for IFNα and from 15 to 25 pg/ml for IFNγ.

We did not detect any variation for IFNβ mRNA, *in vivo* as well as *in vitro*. Lack of IFNβ induction is already well documented and has been suggested to explain, at least partly, why chickens are less sensitive to the deleterious effects of LPS (4). Lack of the intracellular adaptor protein TRAM may explain why TLR4 and TLR2 activation does not result in IFNβ gene transcription (4). In mammals, this protein has indeed been demonstrated to be required to allow TLR2 and TLR4 to transduce through a MyD88 independent pathway (35, 36). This is consistent with our *in vitro* results, as IFNα and IFNγ are regulated by NF-κB and ERK in neutrophils. In monocytes a similar regulation is observed for IFNγ whereas we have only been able to identify NF-κB as transcriptional regulator for IFNα. Both NF-κB and ERK can be activated after TLR2 and TLR4 activation through the MyD88 dependent pathway (37).

ChIFNα is strongly induced in response to a number of viral infections, such as influenza A virus and Newcastle disease virus (38). In addition, a putative binding site for NF-κB has been found in the promoter of this gene (39). *In vitro*, chIFNα strongly hampers the growth of Marek's disease virus, infectious bursal disease virus, vesicular stomatitis virus and infectious bronchitis virus (38, 40). These antiviral activities are not limited to *in vitro* systems since chIFNα has been shown to inhibit the replication of influenza virus (H9N2) infection *in ovo* as well as *in vivo* (40, 41). Moreover, as observed in our study, IFNα allows interferon-stimulated genes to be transcribed (38). Among them is OAS, whose activation results in the cleavage of viral RNA transcripts and host RNAs (38). In our work, its transcription is tuned on in monocytes and heterophils *in vivo*. Consistent with its regulation by IFNα and with the plasmatic concentrations of IFNγ we observed, the major variations in fold changes are at day 1 and day 2 according to the cellular type. However, as two alleles of the OAS gene with different anti-viral activities have been described in chicken (38), further studies may be of interest to identify which one is present in chickens with the Ross 308 genetic background.

In addition, IFNα has been described to promote murine NK cells expansion by protecting them from fratricide (42). As we observed IFNγ release *in vivo*, and as in mammals activated NK cells produce IFNγ, we cannot rule out that NK cells may contribute in our model to part of the IFNγ release. It would thus be of interest to further investigate whether IFNα may act similarly as in mammals, thus contributing to high IFNγ plasma concentrations (42). Moreover a direct action of the ulvan extract on NK lymphocytes could also be considered as the TLR expression pattern on NK cells has not been described so far (43). This would however require the ulvan extract to cross the intestinal barrier and reach the bloodstream; this may be possible due to its low molecular weight but needs formal demonstration.

As in mammals, chIFNγ is essential for host defense against intracellular pathogens and a hallmark of Th1 immunity (38). The reported biological activity of chIFNγ was similar to its mammalian counterparts including induction of MHC class I and class II to allow antigen presentation (37). In addition, chIFNγ also tightly regulates the production of NO (38) and anti-viral activity against vesicular stomatitis virus, infectious bursal disease virus, Newcastle disease virus (44, 45). Moreover an autocrine loop may occur in our model, as IFNγ priming of avian heterophils upregulates the expression of inflammatory cytokines, including IL1β, IFNγ, IL8 (22). It would in this case be of short duration, as plasma levels of IL1β were very close to the levels of control animals as early as day 2 and as IFNα and IFNγ concentrations also decline at day 2. Moreover, Andersen al recently reported that in contrast to what is observed in mammals, IFNγ in chicken may also be produced by CD3+ TCRγδ cells (2). This leads us to question whether these cells may be activated by ulvans and will require further experiments to be performed.

Moreover, St Paul et al reported IL1β, IFNα, and IFNγ genes to be more transcribed in the spleen of birds that received a mixture of TLR4 and TLR21 agonists (46). This also needs to be analyzed in our model of oral administration, as well as the contribution of Bu1+ B cells and tonsils cells as they express TLR2 and TLR4 (18, 43, 47).

Finally, we cannot rule out that ulvan also act through the modulation of GALT functions. TLR2 and TLR4 expression has been described throughout the avian digestive tract despite the fact that the subpopulations were not purified (43). In mouse, TLR4 activation on enterocyte or on intra-epithelial γδ lymphocytes regulates intestinal permeability. If this also happens in chicken, this could modify the ulvan blood transit (48, 49).

## Conclusion

We report for the first time that ulvan activates TLR4 and TLR2 on avian heterophils and monocytes. *In vitro*, we have also demonstrated that the signaling pathways of these receptors display differences in their ability to induce heterophils degranulation and oxidative burst. In addition, the transcriptional regulation of several cytokines genes was tuned on in the two cellular types, partly in a cell specific manner. *In vivo*, when given *per os*, the ulvan extract stimulates, directly and/or indirectly, key players of the chicken innate immune system, i.e., heterophils and monocytes. The release *in vivo* of IL1β, IFNα, and IFNγ suggest a Th1 orientation of the immune response. The protective effect ulvans may confer will however also be dependent on the pathogens' virulence and on their adaptability, as for any prophylactic agent.

Further translational and fundamental studies are necessary to fully understand its mode of action.

## Author contributions

NG, FB, ML, and PC contributed to the conception and design of the study, including the protocol for the ethical committee. CG and PC prepared the ulvan extract. FB, CG, OM, BQ, and ML prepared and performed the *in vivo* trial. NG performed the statistical and biological analysis and wrote the first draft of the manuscript. CG, FB, and PC wrote sections of the manuscript. All authors contributed to manuscript revision, read and approved the submitted version.

### Conflict of interest statement

FB, CG, ML, and PC were employed by company Amadeite. The remaining authors declare that the research was conducted in the absence of any commercial or financial relationships that could be construed as a potential conflict of interest.

## Supplementary material

Supplemental Figure 1 RMN proton analysis. The samples were dissolved on 99.97% atome D_2_O and subjected to RMN proton analysis. The RMN proton spectrum was registered at 298 K on a Bruker Avance 500 spectrometer with a inversed cryogenic probe 5 mm _1_H/_13_C/_15_N TCI. The isotopic shifts were referenced with respect to an external standard (trimethylsilypropionic acid). No suppression of the HOD signal was performed.

Supplemental Figure 2 Lipids were extracted with CHCl_3_/MeOH (2/1; v/v) and the amount of palmitic acid quantified using GC-FID (gas chromatography with flame ionization detector) according to Le Croizier et al. (10). Fatty acids were identified by comparison of their retention time with the ones of commercial standards. Comparing the fatty acids area with the one of the internal standard (C23:0) allowed their amount to be determined.

Supplemental Table 1 Primers sequences and relative references.

Supplemental Table 2 Fold change that do not vary statistically in heterophils *in vitro*.

Supplemental Table 3 Fold change that do not vary statistically in monocytes *in vitro*.

Supplemental Table 4 Animals weight during the three *in vivo* trials. ^*^The company does not provide standard deviation values.

Supplemental Table 5 Fold change that do not vary statistically in heterophils *in vivo*.

Supplemental Table 6 Δ-Ct values for all the studied genes in heterophils during the three *in vivo* trials.

Supplemental Table 7 Fold change that do not vary statistically in monocytes *in vivo*.

Supplemental Table 8 Δ-Ct values for all the studied genes in monocytes during the three *in vivo* trials.
