# Supplementary figures and images for: Ulvan Activates Chicken Heterophils and Monocytes Through Toll-Like Receptor 2 and Toll-Like Receptor 4
Source: Front Immunol. 2018 Nov 23;9:2725. doi: 10.3389/fimmu.2018.02725 (PMC6265352; doi:10.3389/fimmu.2018.02725)

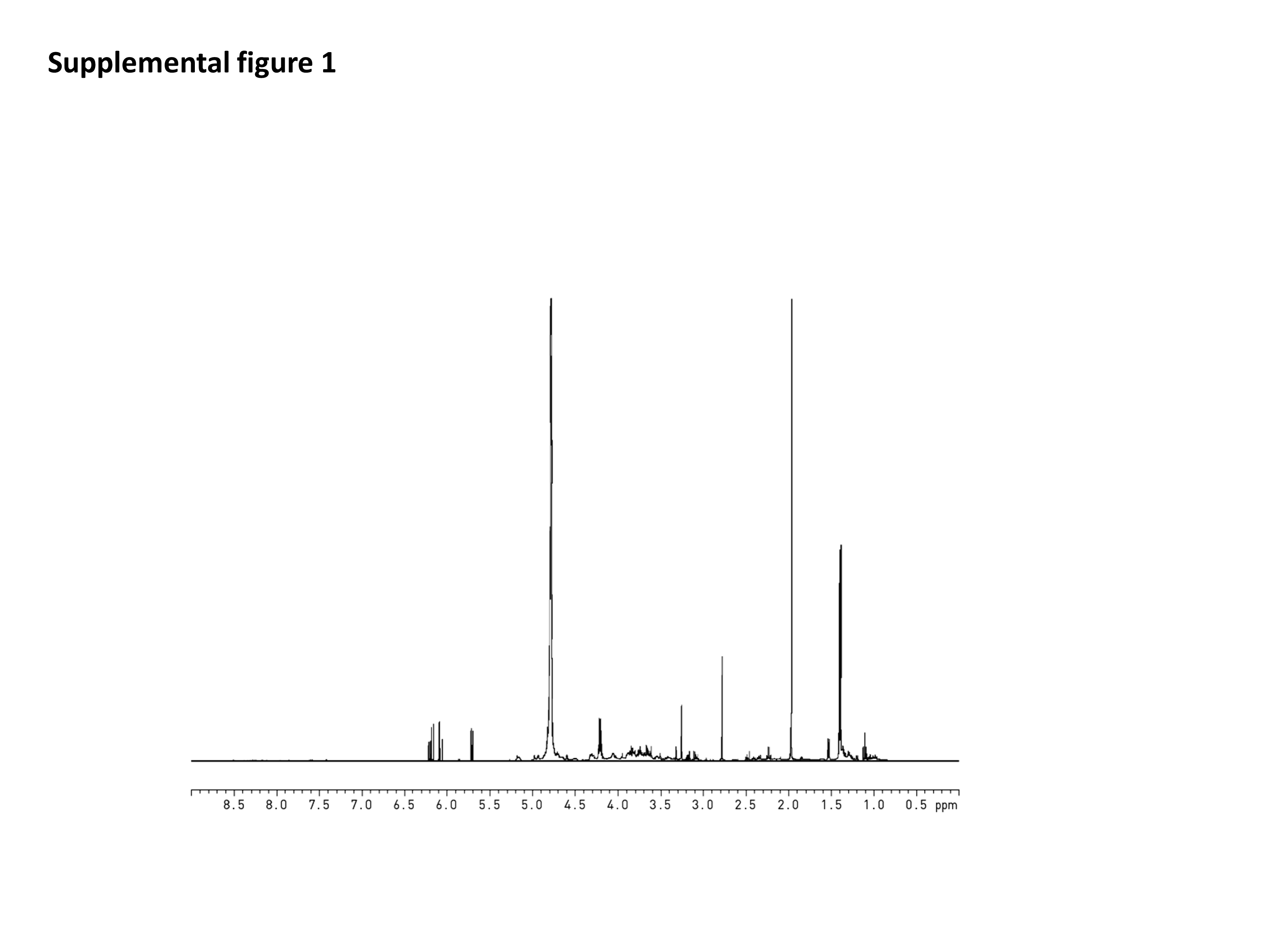

Supplement: Supplemental Figure 1 — RMN proton analysis. The samples were dissolved on 99.97% atome D2O and subjected to RMN proton analysis. The RMN proton spectrum was registered at 298 K on a Bruker Avance 500 spectrometer with a inversed cryogenic probe 5 mm 1H/13C/15N TCI. The isotopic shifts were referenced with respect to an external standard (trimethylsilypropionic acid). No suppression of the HOD signal was performed. [file Image_1.TIF]

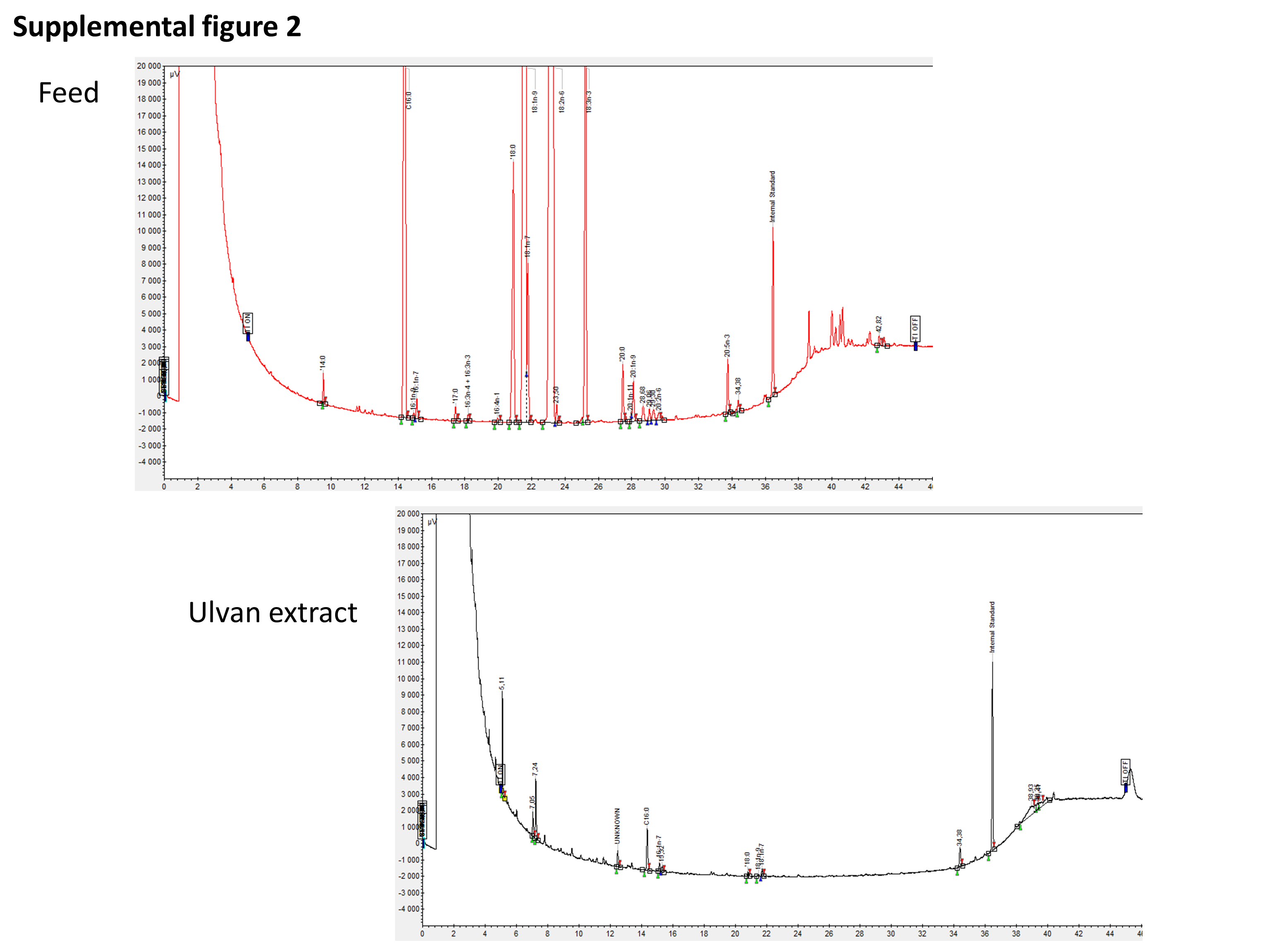

Supplement: Supplemental Figure 2 — Lipids were extracted with CHCl3/MeOH (2/1; v/v) and the amount of palmitic acid quantified using GC-FID (gas chromatography with flame ionization detector) according to Le Croizier et al. (10). Fatty acids were identified by comparison of their retention time with the ones of commercial standards. Comparing the fatty acids area with the one of the internal standard (C23:0) allowed their amount to be determined. [file Image_2.TIF]
